# Similarities and differences in constipation phenotypes between *Lep* knockout mice and high fat diet-induced obesity mice

**DOI:** 10.1371/journal.pone.0276445

**Published:** 2022-12-22

**Authors:** Ji Eun Kim, Ayun Seol, Yun Ju Choi, Su Jin Lee, You Jeong Jin, Yu Jeong Roh, Hee Jin Song, Jin Tae Hong, Dae Youn Hwang

**Affiliations:** 1 Department of Biomaterials Science (BK21 FOUR Program), College of Natural Resources & Life Science, Pusan National University, Miryang, Korea; 2 College of Pharmacy, Chungbuk National University, Chungju, Korea; 3 Life and Industry Convergence Research Institute/Laboratory Animal Resources Center, College of Natural Resources & Life Science, Pusan National University, Miryang, Korea; University of Nevada School of Medicine, UNITED STATES

## Abstract

CRISPR-Cas9-mediated leptin (*Lep*) knockout (KO) mice exhibited prominent phenotypes for constipation, even though they were not compared with other model animals. This study compared the stool excretion, gastrointestinal motility, histological structure, mucin secretion, and enteric nerve function in *Lep* KO and high fat diet (HFD)-treated mice to determine if there were differences in their phenotypes for constipation. Most obesity phenotypes, including fat weight, adipocyte size, expression of lipolytic proteins (HSL, perilipin, and ATGL), and glucose concentrations, were detected similarly in the *Lep* KO and HFD-treated mice. They showed a similar decrease in the excretion parameters, including the stool number, weight, and water content, while the same pattern was detected in the gastrointestinal motility and intestinal length. A similar decrease in the mucosal layer thickness, muscle thickness, ability for mucin secretion, and expression of water channel (aquaporin 3 and 8) genes was detected in the mid-colon of the *Lep* KO and HFD-treated mice, but the alteration rate in some levels was greater in the HFD-treated group than the *Lep* KO mice. On the other hand, the levels of c-kit, nNOS, NSE, and PGP9.5 expression for the enteric neurons and intestitial cells of Cajal (ICC) were remarkably lower in the mid-colon of the HFD-treated mice than in the *Lep* KO mice, but the level of most proteins in both groups remained lower than those in the control group. A similar alteration pattern in the expression of muscarinic acetylcholine receptors (mAChRs) and serotonin receptors was detected in the *Lep* KO and HFD-treated mice. These results suggest that most phenotypes for obesity-induced constipation were similarly detected in the *Lep* KO and HFD-treated mice, but there was a difference in the regulatory function of the enteric nervous system (ENS).

## Introduction

Obesity is an important risk factor for health problems, including diabetes, cancer, fatty liver, hyperlipidemia, hypertension, stroke, and arthritis [1, 2]. Several studies have examined the correlation between obesity and constipation, but there was a difference in their proportion. Approximately 60% of adults with functional constipation have a higher body mass index (BMI, 26.5) than the community mean. In contrast, the frequency of constipation is 4.5 times higher in obese patients than in normal-weight people [3, 4]. The prevalence of chronic constipation and fecal soiling was higher in the population of obese children, but the rate of the increase was different [2–6]. In particular, HFD-induced obesity was suggested to be one of the main causes of constipation because several phenotypes for this disease were detected during the pathogenesis of obesity. C57BL/6 mice given a Western diet (WD) for 9 and 12 weeks showed a decrease in nitrergic myenteric neurons linked to delayed colonic transit [7]. Significant phenotypes for constipation were also detected in the C57BL/6 mice fed a HFD for eight weeks. They showed an increase in the total gastrointestinal (GI) transit time and colon transit time and a decrease in the amount of colonic mucus [8]. A similar decrease in the fecal amount was observed in the NSY/HOS mice fed a HFD for four weeks [9]. On the other hand, the level of fecal mucin was maintained in the SD rats treated with a low-fat diet (LFD) and HFD for three weeks [10]. Therefore, it is essential to compare the constipation phenotypes in various models, while comparing the molecular mechanisms of obesity and constipation.

Obesity is associated with changes in the motility of the GI tract, including the esophagus, stomach, small intestine, and colon [11]. Among them, a disruption of colonic motility can occur during various digestive diseases, such as irritable bowel syndrome (IBD), constipation, diarrhea, and bloating, because the stimulation of colonic motility leads to an increase in colonic tone, migratory long spike bursts, and contraction for the propagation and segmentation of stools [12, 13]. A high-fat meal induces a strong postprandial colonic response in normal individuals, and stimulates retrograde phasic contractions that cause a delay in colonic transit [14]. In addition, obese patients showed significant changes in the colonic sensory and motor neuronal function, including an increase in pain threshold, decreased compliance to an inflated barostat, and a short transit time, whereas the prevalence of diarrhea was increased in the same patients [15]. Furthermore, the role of serotonin was identified in the correlation between obesity and colonic motility. The serotonin availability and enterochromic cell number were lower in HFD-induced obese rats with constipation [16]. On the other hand, changes in various constipation phenotypes, including the stool parameters, histological structure, and ENS regulation, were not compared directly in the obesity-induced constipation model.

This study compared the pathological symptoms and molecular mechanism of constipation in *Lep* KO and HFD-treated mice by examining the stool parameters, histopathology, GI transit, mucin secretion, and ENS regulation. These results provide the first evidence that HFD-induced obesity mice show more severe damage to ENS regulation among the constipation phenotypes than *Lep* KO mice, but the other phenotypes were similar.

## Materials and methods

### Animal study

The animal protocol to characterize novel phenotypes was reviewed and approved by the Pusan National University-Institutional Animal Care and Use Committee (PNU-IACUC) based on the ethical procedures for scientific care (Approval Number PNU-2019-2293; PNU-2018-1870). All mice were maintained at the Pusan National University-Laboratory Animal Resources Center, accredited by the Korea Food and Drug Administration (KFDA) (Accredited Unit Number-000231) and the Association for Assessment and Accreditation of Laboratory Animal Care (AAALAC) International (Accredited Unit Number; 001525). The mice were provided access to a standard irradiated chow diet (Samtako BioKorea Inc., Osan, Korea) and water *ad libitum*. Throughout the experiment, the mice were maintained in a specific pathogen-free (SPF) state under a strict light cycle (on at 08:00 h; off at 20:00 h) at 23±2°C and 50±10% relative humidity.

The wild-type (Con) and *Lep* KO (C57BL/6-*Lep*^em1Shwl^/Korl) mice were kindly provided by the Department of Laboratory Animal Resources in the National Institute of Food and Drug Safety Evaluation (NIFDS, Chungju, Korea). The *Lep* KO mice were identified genomically from the DNA extracted from the tails of three-week-old founder mice by DNA-PCR analysis, as described elsewhere [17, 18]. The *Lep* KO mice with the constipation phenotypes at 16 weeks of age (n = 7) and the Con mice at the same age (n = 7) were used in the comparative studies. The eight-week-old C57BL/6 male mice were provided by Samtako Bio-Korea Inc. (Osan, Korea) and fed with a HFD containing 60% kcal fat from Research Diets (cat. no. D12492; Research Diets, Inc., New Brunswick, USA) for eight weeks. Subsequently, the constipation phenotypes were analyzed in the HFD-induced obesity mice (n = 7). After the final treatment, several tissue samples were acquired from the mice euthanized using CO_2_ gas and stored in Eppendorf tubes at −70°C until assay.

### Confirmation of the obesity phenotypes, and measurement of body and fat weight

At the last day of the experiment, the mice were photographed with a digital camera, and their morphological features were compared. The body weight of the Con, *Lep* KO, and HFD-treated mice was measured using an electronic balance (Mettler Toledo, Greifensee, Switzerland), according to the KFDA guidelines. In addition, the weights of retroperitoneal and epididymal fat collected from the sacrificed mice in the subset groups were determined using the same method to measure the body weights.

### Measurement of feeding behavior and excretion parameters

The total weight of foods and water volume were measured in the Con, *Lep* KO, and HFD-treated mice at 9 a.m. on the 5^th^ day using an electrical balance. The average food intake and water consumption were calculated as the difference between the amount of feed (water) supplied and the amount of feed (water) remaining.

All mice were bred individually in metabolic cages to provide uncontaminated stool and urine samples (Daejong Instrument Industry Co., LTD, Seoul, Korea). Briefly, the stools eliminated from each mouse were collected at 9 a.m. and weighed in duplicate using an electric balance. The total number of stools was counted twice per mouse, and their moisture contents were also analyzed as follows:

Stoolmoisturecontent=(A−B)/A×100

where A is the weight of fresh stools immediately collected from the metabolic cage, and B is the weight of stools after drying at 60°C for 24 h. The volume of urine collected from metabolic age at the same time was measured using a cylinder.

### Determination of glucose concentration

After fasting for 18 h, whole blood was collected from the abdominal veins of all mice. The blood samples were incubated for 30 min at room temperature in serum separating tubes (BD Biosciences, Franklin Lakes, NJ, USA). The serum was obtained by centrifugation at 1,500 xg for 15 min. The serum glucose concentration was analyzed using the automatic chemical analyzer (BS-120 Chemistry Analyzer; Mindray, Shenzhen, China).

### Measurement of GI transit ratio and total GI tract length

The GI transit ratio was measured using the method described elsewhere [19]. Briefly, all mice of the subset groups were fasted for 18 h before the experiment, but were given access to water *ad libitum*. Each mice in the subset group was fed 0.3 mL of charcoal meal (3% suspension of activated charcoal in 0.5% aqueous methylcellulose) (Sigma–Aldrich Co., St. Louis, MO, USA). After 30 min of administration, the mice were euthanized with CO_2_, and the GI tract was collected from the abdominal cavity. The intestinal charcoal transit ratio was calculated as follows:

Charcoaltransitratio(%)=[(totalsmallintestinelength–transitdistanceofcharcoalmeal)/totalsmallintestinelength)]×100

The total GI tract length from the stomach to the anus was also measured in duplicate.

### Histopathological analysis

The livers, fats, and mid colons collected from the mice were fixed in 10% formalin for 48 h. The samples were then embedded in paraffin wax, cut into 4 μm thick sections, and stained with hematoxylin and eosin (H&E, Sigma–Aldrich Co.). The mucosa and muscle layer thicknesses in the mid colon sections were then analyzed by optical microscopy (Leica Microsystems, Wetziar, Germany). The adipocyte size and lipid droplet number were examined microscopically in the fat and liver sections at 400× magnification. Prof. Beum Seok Han, a pathologist at the Department of Pharmaceutical Engineering, Hoseo University (Asan, Chungcheongnamdo), Korea, characterized the pathological features of each tissue sample.

Mucin staining analysis was achieved by fixing the mid colons collected from the mice of all the subset groups in 10% formalin for 48 h, embedding the samples in paraffin wax, and sectioning them into 4 μm thick slices, which were then deparaffinized with xylene and rehydrated. The mounted tissue sections were rinsed with distilled water and stained using an Alcian Blue Stain kit (IHC WORLD, Woodstock, MD, USA). The stained patterns in the mid colon sections were observed by optical microscopy.

### Western blotting analysis

The total proteins were collected from the mid colons or livers of each mice using the Pro-Prep Protein Extraction Solution (Intron Biotechnology Inc., Seongnam, Korea) according to the manufacturer’s protocol. The acquired proteins were centrifuged at 13,000 rpm at 4°C for 5 min, and the total protein concentrations were determined using a SMARTTM Bicinchoninic Acid Protein assay kit (Thermo Fisher Scientific Inc., Wilmington, MA, USA). The proteins (30 μg) were subjected to 4–20% sodium dodecyl sulfate-polyacrylamide gel electrophoresis (SDS-PAGE) for 3 h, and the resolved proteins were transferred to a nitrocellulose membrane for 2 h at 40 V. The membranes were then probed overnight with the following primary antibodies at 4°C: anti-nNOS (Abcam Com., Cambridge, UK), anti-NSE (Abcam Com.), anti-MLC (Abcam Com.), anti-p-MLC (Abcam Com.), anti-HSL (Cell Signaling Technology Inc., Danvers, MA, USA), anti-p-HSL (Cell Signaling Technology Inc.), anti-perilipin (Cell Signaling Technology Inc.), anti-p-perilipin (Cell Signaling Technology Inc.), anti-ATGL (Cell Signaling Technology Inc.), anti-Gα (Abcam Com.), anti-mAChR M2 (Alomone Labs, Jerusalem, Israel), anti-mAChR M3 (Alomone Labs), anti-PKC (Cell Signaling Technology Inc.), anti-p-PKC (Cell Signaling Technology Inc.), anti-PI-3K (Cell Signaling Technology Inc.), anti-p-PI3K (Cell Signaling Technology Inc.), or anti-β-actin (Sigma–Aldrich Co.). The resulting membranes were washed with a washing buffer (137 mM NaCl, 2.7 mM KCl, 10 mM Na_2_HPO_4_, 2 mM KH_2_PO_4_, and 0.05% Tween 20), followed by incubation with 1:1,000 diluted horseradish peroxidase-conjugated goat anti-rabbit IgG (Zymed Laboratories, South San Francisco, CA, USA) for 2 h at room temperature. The blots were then developed using a Chemiluminescence Reagent Plus kit (Pfizer Inc., Gladstone, NJ, USA). The signal images of each protein were then acquired using a digital camera (1.92 MP resolution) of the FluorChem^®^ FC2 Imaging system (Alpha Innotech Corporation, San Leandro, CA, USA). The protein densities were semi-quantified using the AlphaView Program, version 3.2.2 (Cell Biosciences Inc., Santa Clara, CA, USA).

### qRT-PCR analysis

The total RNA was isolated from the mid colons of the mice (n = 4) in each subset group using an RNA Bee solution (Tet-Test, Friendswood, TX, USA). After homogenizing the frozen mid colon tissue, the RNA was isolated by centrifugation at 15,000 rpm for 15 min, and the concentration was measured using a Nano Drop Spectrophotometer (Allsheng, Hangzhou, China). The total RNA (5 μg) from the mid colon tissue was annealed with 500 ng of oligo-dT primer (Thermo Fisher Scientific Inc.) at 70°C for 10 min to select the mRNA of each gene. The complementary DNA (cDNA) was synthesized using the Invitrogen Superscript II reverse transcriptase (Thermo Fisher Scientific Inc.). qPCR was performed using the cDNA template obtained (2 μL) and 2× Power SYBR Green (6 μL; Toyobo Life Science, Osaka, Japan) containing the following specific primers: Lep, sense primer 5′-AGC TGC AAG GTG CAA GAA GAA-3′ and antisense primer 5′-GGA ATG AAG TCC AAG CCA GTG AC-3′; mAChR M1, sense primer 5′–TCCTC TCCCA ACCCA TCATC–3′ and antisense primer 5’–GACCG TGACA GGGAG GTAGA AG–3′; mAChR M2, sense primer 5′–CTGCG TGGGT CCTTT CCTT–3′ and antisense primer 5’–CCTCA CCCCT ACGAT GAACT G–3′; mAChR M3, sense primer 5′–CAGGG CCATC TATTC CATTG TC–3′ and antisense primer 5’–GTTAT CTGAG GACGG TAGCT TGGT–3′; mAChR M4, sense primer 5′–TGGTG AGCCT CAAGG CACTA–3′ and antisense primer 5’–ATGGG ATCTG GATGG ACACT TT–3′; mAChR M5, sense primer 5′–CCTGG TCATC CTCCC GTAGA–3′ and antisense primer 5’–GCCTT TTCCC AGTCA GCACT T–3′; 5-HT 2AR, sense primer 5′–CCGGG AGCCT CTTGA TACAG–3′ and antisense primer 5’–AGCCC CTCTC AAAGT CACAC A–3′; 5-HT 2BR, sense primer 5′–GCAGA TTTGC TGGTT GGATT G–3′ and antisense primer 5′–GGCCA TATAG CCTCA AACAT GAT–3′; 5-HT 3AR (5-hydroxytryptamine receptor 3A) sense primer 5′–CTGAG GCCCT CCCAC ATCT–3′ and antisense primer 5′–GGAAA GGAAC AAGGC CAACA–3′; 5-HT 3BR, sense primer 5′–TGCCG AGGAG TCTAG ATTGT ACCT–3′ and antisense primer 5′–ACCCG ATGCT CCTGA TGGA–3′; β-actin sense primer 5′–ACGGC CAGGT CATCA CTATT G–3′ and antisense primer 5′–CAAGA AGGAA GGCTG GAAAA GA–3′. qRT-PCR was performed for 40 cycles using the following sequence: denaturation at 95°C for 15 s, followed by annealing and extension at 70°C for 60 s. The fluorescence intensity was measured at the end of the extension phase of each cycle. The threshold value for the fluorescence intensities of all the samples was set manually. The reaction cycle at which the PCR products exceeded this fluorescence intensity threshold during the exponential phase of PCR amplification was considered the threshold cycle (Ct). The expression of the target gene was quantified relative to that of the housekeeping gene β-actin based on a comparison of the Cts at constant fluorescence intensity according to Livak and Schmittgen’s method [20]. The relative quantification formula was the 2−ΔΔCt method proposed by Livak and Schmittgen.

### Statistical analysis

The statistical significance was evaluated using the One-way Analysis of Variance (ANOVA) (SPSS for Windows, Release 10.10, Standard Version, Chicago, IL, USA) followed by a Tukey post hoc t-test for multiple comparisons. All values are expressed as the means ± SD. A p-value (p < 0.05) was considered significant.

## Results

### Similarities and differences in the obesity phenotypes between the *Lep* KO and HFD-treated mice

**Fig 1:**
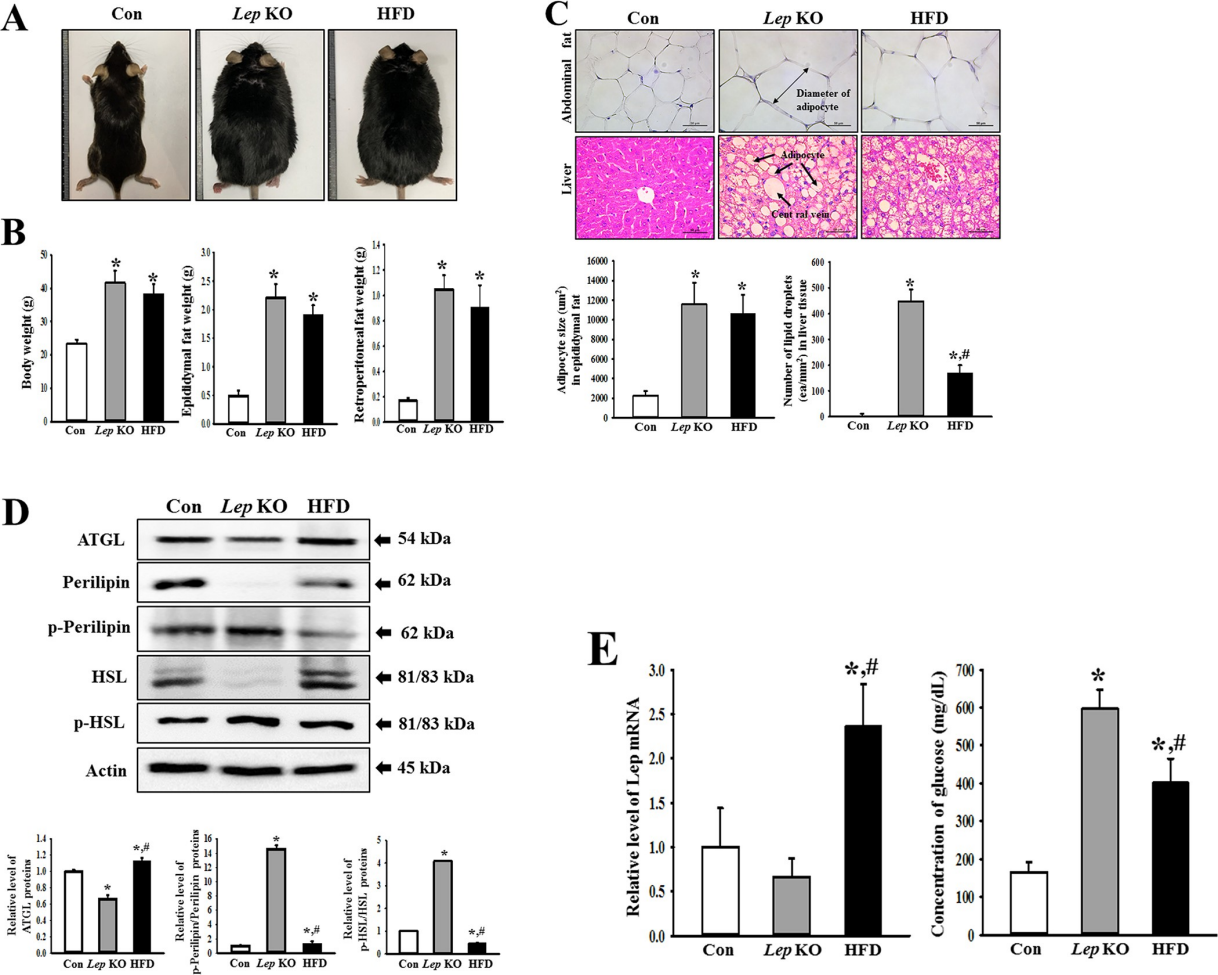
Obesity phenotypes of *Lep* KO and HFD-treated mice. (A) Morphology of the animals. The animals were photographed using a digital camera after anesthesia. (B) Body and fat weights. Five to six mices were selected from each group, and their weights were assayed in duplicate using an electric balance. (C) Histological structure of fat and liver tissue. After hematoxylin and eosin (H&E) staining of the fat and liver tissue, their histopathological features were observed at 400× magnification using an optical microscope. The area of each adipocyte in fat and the number of lipid droplets in the liver were measured using the Image J program. Five to six mices per group were used in histological analysis, and each parameter was measured in duplicate on two different slides. (D) Expression of the lipogenic proteins. The levels of ATGL, Perilipin, p-Perilipin, HSL, and p-HSL expression were measured by Western blot analysis using the specific primary antibodies and HRP-labeled anti-rabbit IgG antibody. Three to five mices per group were used to prepare the total tissue homogenate, and Western blot analyses were assayed in duplicate in each sample. (E) Expression of Lep mRNA and glucose concentration. The relative levels of Lep mRNA were determined in the liver tissue using RT-qPCR, while the glucose concentration was measured in the serum of whole blood. The data are reported as the mean ± SD. * indicates p < 0.05 compared to the Con group. Abbreviations: Con, Control group; *Lep* KO, *Leptin* knockout mice; HFD, High fat diet; ATGL, Adipose triglyceride lipase; HSL, Hormone-sensitive lipase.

The obesity phenotypes in the *Lep* KO and HFD-treated mice were compared to confirm the successful induction of the obesity phenotype. This was achieved by analyzing the changes in the appearance, body and fat weight, histological structure, lipogenic proteins, Lep expression, and glucose concentration in the *Lep* KO and HFD-treated mice. These two models were significantly fatter than the Con mice, but there was no difference between the two obesity models (Fig 1A). The body weights increased remarkably by 77% and 63% in the *Lep* KO and HFD-treated mice, respectively, compared to those in the Con mice, even though there are no significant differences between the two obesity models (Fig 1B). In addition, the weights of the retroperitoneal and epididymal fat were enhanced remarkably in the *Lep* KO and HFD-treated mice (Fig 1B). Similar alterations were observed in the H&E stained fat and liver sections. The *Lep* KO and HFD-treated mice showed a significant increase in the adipocyte size in fat tissue and the number of lipid droplets in the liver tissue compared to the Con mice (Fig 1C). On the other hand, some significant differences were measured in the expression of lipogenic proteins. The level of perilipin and HSL phosphorylation was 13 and 4.2 times higher in the *Lep* KO mice, respectively, than in the HFD-treated mice. These levels in the two obesity models were maintained at high levels compared to the Con mice. In contrast, the level of ATGL expression was lower in the *Lep* KO mice than in the HFD-treated mice (Fig 1D). Moreover, a significant increase in the glucose concentration was detected in the *Lep* KO and HFD-treated mice compared to the Con mice, whereas an increase in the expression level of Lep mRNA was observed only in HFD-treated mice (Fig 1E). These results suggest that the *Lep* KO and HFD-treated mice used in the present study have similar phenotypes for obesity except for the regulation of lipogenic proteins and LEP expression.

### Similarity in the excretion parameters and feeding behavior of the *Lep* KO and HFD-treated mice

**Fig 2:**
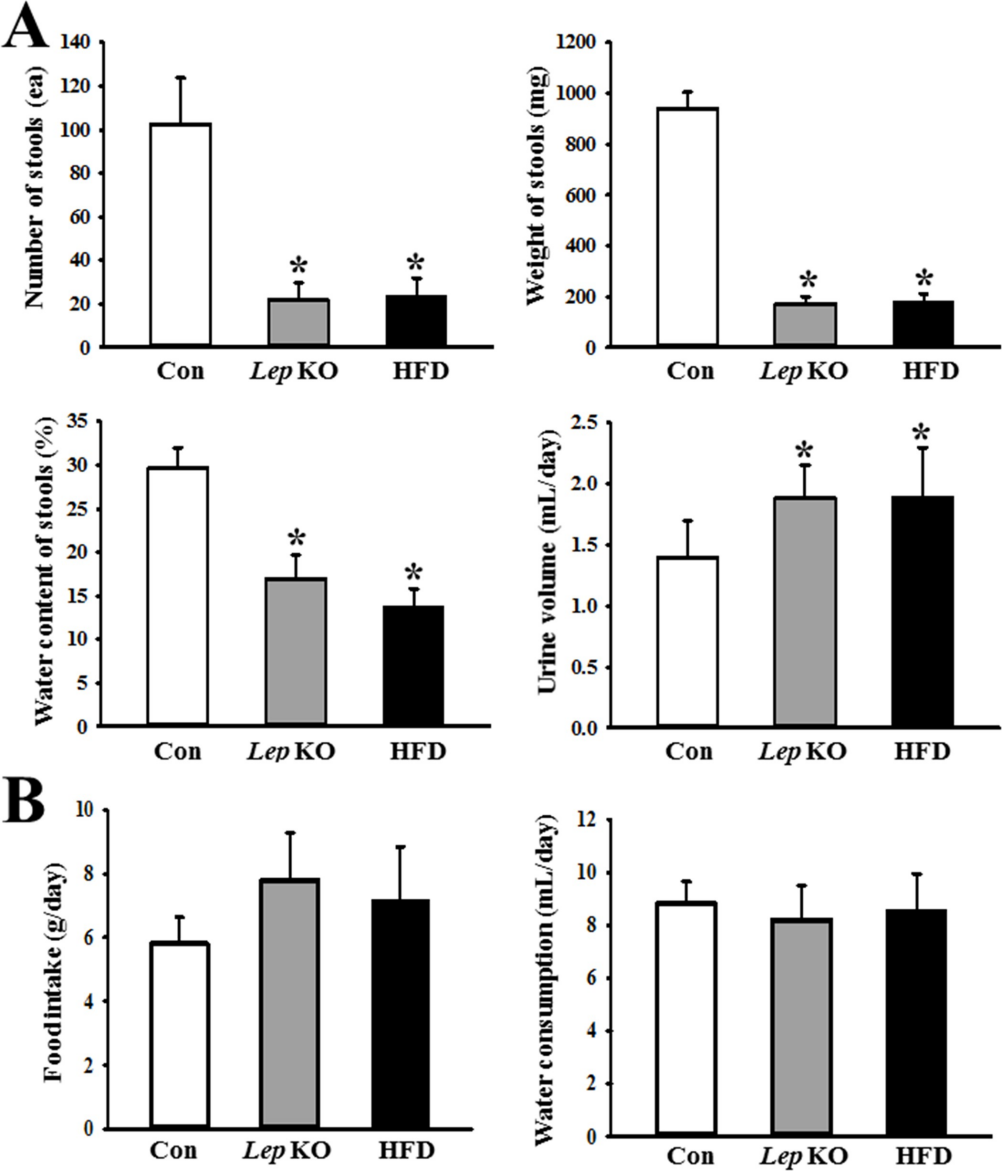
Excretion parameters and feeding behavior. (A) The levels of the four excretion parameters, including number of stools, the weight of stools, water contents of stool, and urine volume, were measured from mice bred in a metabolic cage. Five to six mices per group were used for the stool and urine sample collection, and each parameter was assayed in duplicate. (B) The food intake and water consumption were also calculated using the amount of feed (water) supplied and the amount of feed (water) remaining. The data are reported as the mean ± SD. * indicates p < 0.05 compared to the Con group. Abbreviations: Con, Control group; *Lep* KO, *Leptin* knockout mice; HFD, High fat diet.

Based on previous studies characterizing the decrease in excretion parameters in the *Lep* KO mice, this study examined whether these analyzed phenotypes were similarly detected in the HFD-treated mice. The changes in the number, weight, and water contents of the stools and urine volume in the *Lep* KO and HFD-treated mice were compared. The levels of the three stool parameters (number, weight, and water content) were lower at 79%, 82%, and 43%, respectively, in the *Lep* KO mice than in the Con mice. A similar alteration pattern was observed in the HFD-treated mice. In addition, the urine volume in the *Lep* KO and HFD-treated mice increased by 34% and 35%, respectively (Fig 2A). On the other hand, the food intake and water consumption remained constant in all groups (Fig 2B). Hence, a significant decrease in excretion parameters for constipation was also detected in the *Lep* KO and HFD-treated mice, even though there were no changes in feeding behaviors. Furthermore, these results were confirmed by the Spearman correlation between the body weight and stool parameters in *Lep* KO mice (S1 Fig).

### Similarity in the GI motility and length in the *Lep* KO and HFD-treated mice

**Fig 3:**
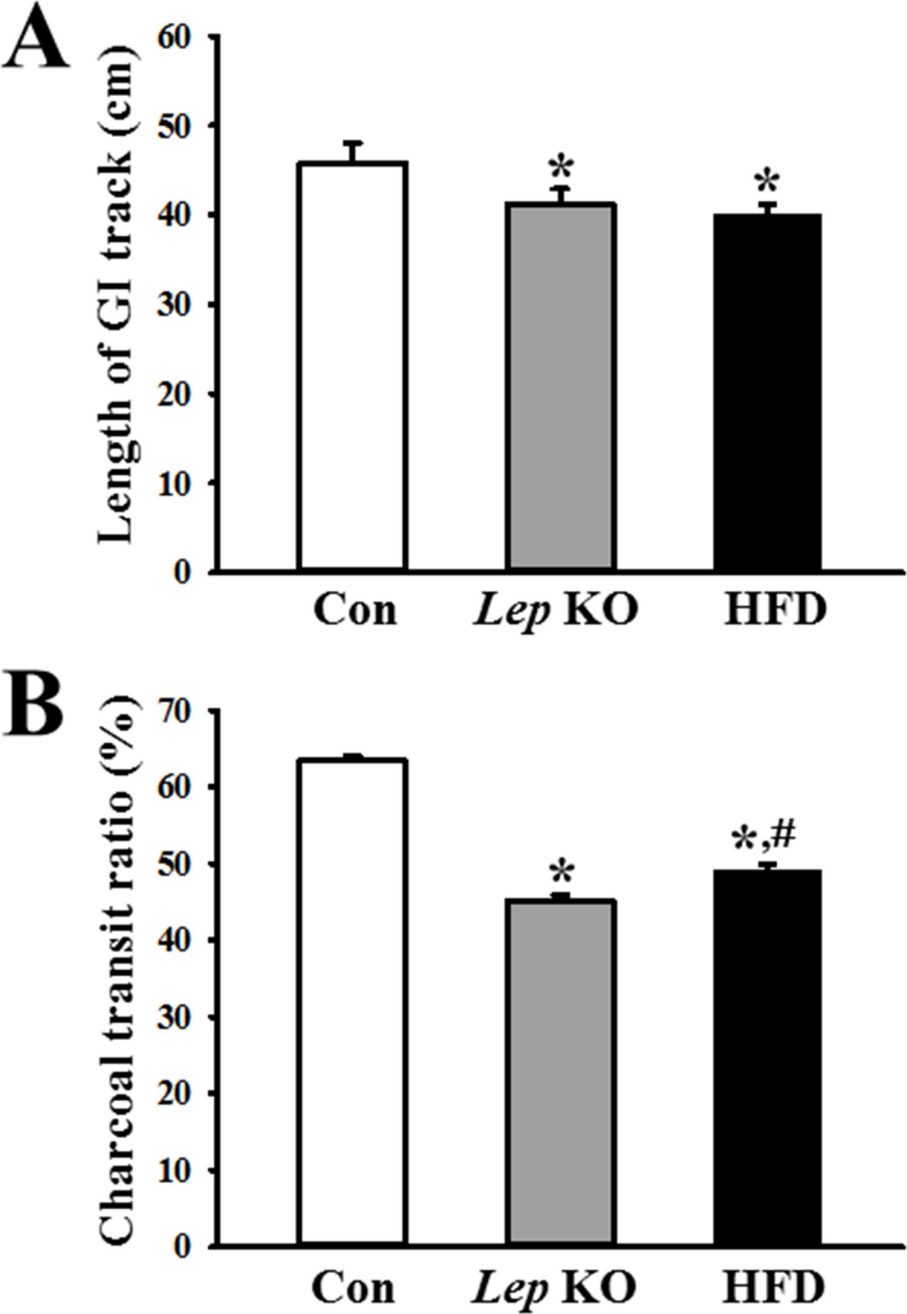
GI tract length and GI transit ratio. (A) Length of the GI tract. Three to five mice per group were used to prepare the GI tract, and their length was measured in duplicate. (B) Charcoal transit ratio. The charcoal meal transit ratio was then calculated using the total length of the intestine and the distance of the charcoal meal. Three to five mices per group were used in the GI transit ratio test, and the charcoal meal transit distance and intestine length were measured in duplicate. The data are reported as the mean ± SD. * indicates p < 0.05 compared to the Con group. Abbreviations: Con, Control group; *Lep* KO, *Leptin* knockout mice; HFD, High fat diet; GI, gastrointestinal.

The changes in the GI motility and length in the *Lep* KO and HFD-treated mice were measured to determine if the decrease in GI motility and length were similar in both groups. The length of the GI tract was lower in the *Lep* KO and HFD-treated mice than in the Con mice, but the difference was not significant (Fig 3A). A similar response in the gastrointestinal ratio was detected. This level was lower in the *Lep* KO and HFD-treated mice than in the Con mice (Fig 3B). Hence, the decreases in GI motility and length were similar in the *Lep* KO and HFD-treated mice.

### Similarities and differences in the histopathological structure of the mid colon between the *Lep* KO and HFD-treated mice

**Fig 4:**
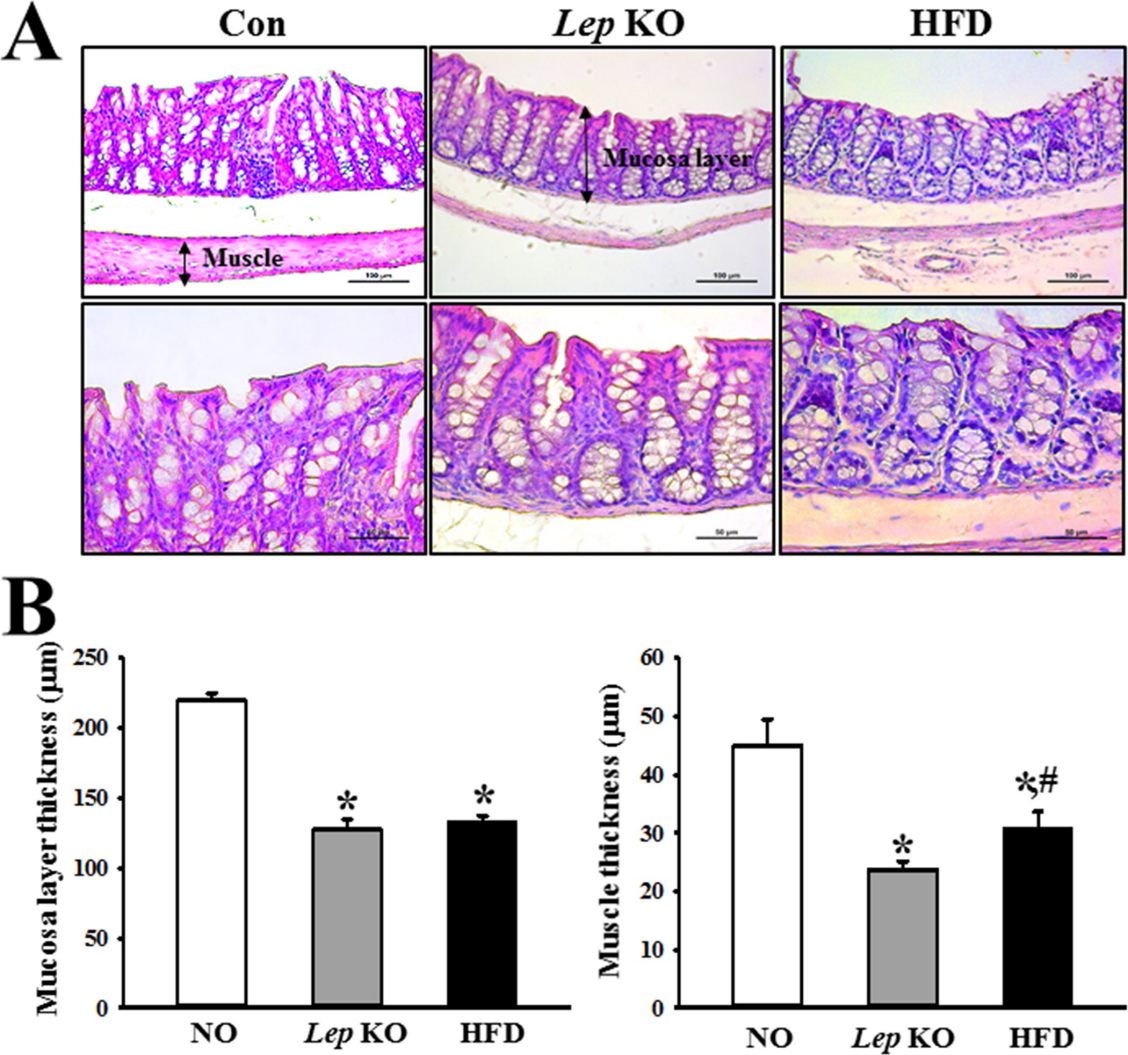
Histological structures of the mid colon. (A) Histological structures of the hematoxylin and eosin (H&E)-stained mid colon. H&E-stained sections of the mid colon from the *Lep* KO mice and HFD-treated mice were observed at 100× and 400× magnification using an optical microscope. (B) The thickness of the mucosal layer and muscle. The histopathological parameters were determined using the Leica Application Suite (Leica Microsystems). Three to five mices per group were used for histological analysis, and each parameter was measured in duplicate on two different slides. The data are reported as the mean ± SD. * indicates p < 0.05 compared to the Con group. Abbreviations: Con, Control group; *Lep* KO, *Leptin* knockout mice; HFD, High fat diet.

The changes in the thickness of the mucosal layer and muscle in the *Lep* KO and HFD-treated mice were measured to determine if the alterations of the histopathological structure were similar in both models. The *Lep* KO and HFD-treated mice showed a significantly lower mucosal layer thickness than the Con mice. On the other hand, there was no difference between the *Lep* KO and HFD-treated mice (Fig 4). In addition, a similar response in muscle thickness was detected. Nevertheless, the decrease rate was greater in the *Lep* KO mice than in the HFD-treated mice (Fig 4). These results suggest that the decrease in mucosal layer thickness is similar in the two obesity models, but the decrease in muscle thickness was greater in the *Lep* KO mice than in the HFD-treated mice.

### Similarities and differences in the mucin secretion ability and water channel expression between the *Lep* KO and HFD-treated mice

**Fig 5:**
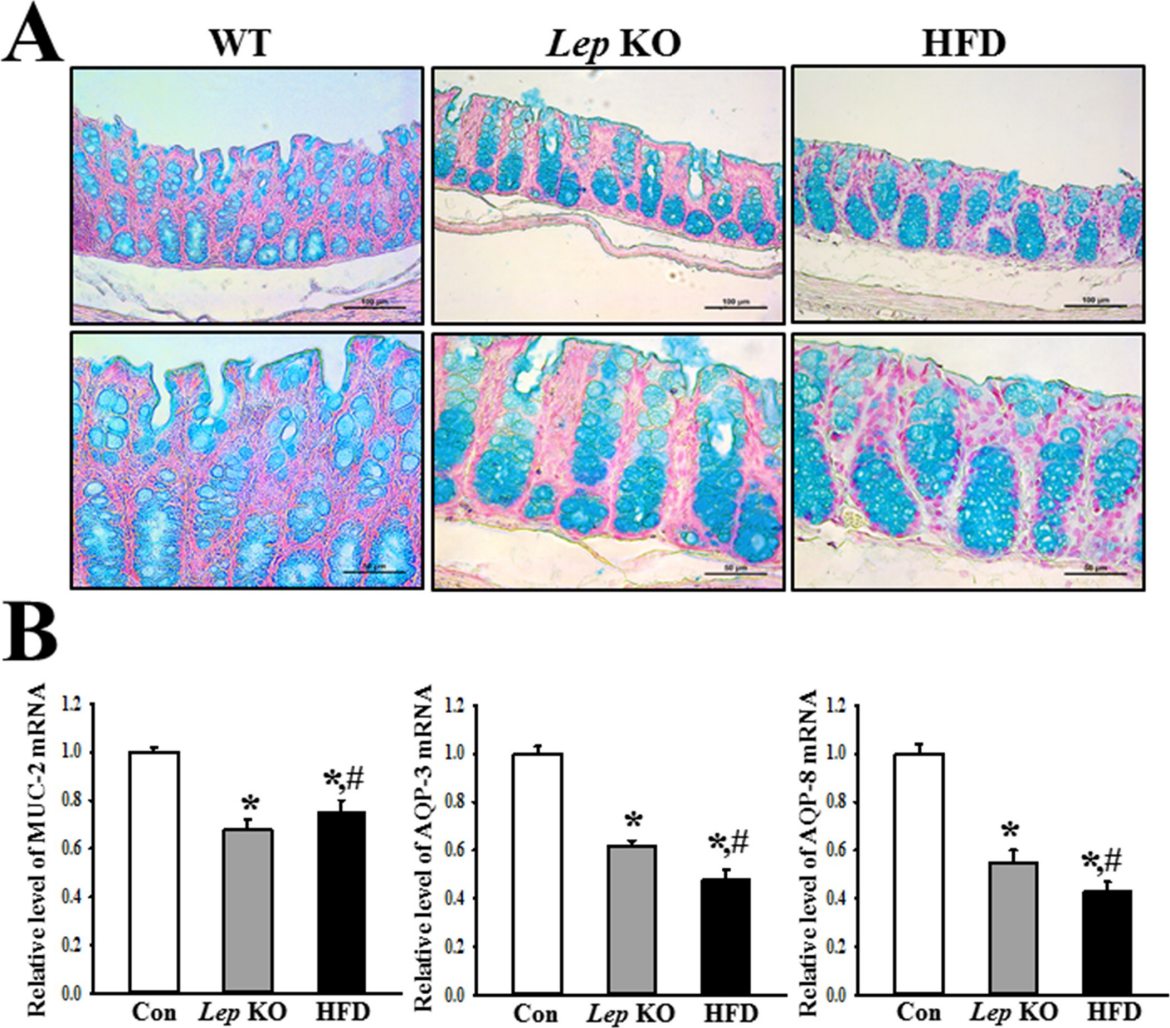
Level of mucin secretion and water channel expression. (A) Level of mucin secretion. Mucin in the mid colon section was stained with alcian blue at pH 2.5. Their images were observed at 400× magnification. Five to six mice per group were used in the slide section, and mucin staining was assessed in duplicate in two different slides. (B) Levels of MUC2, AQP3, and AQP8 expression. The mRNA levels of the three genes were calculated based on the transcript level of β-actin as an endogenous control. Three to five mices per group were used to prepare the total RNA, and RT-qPCR analyses were assayed in duplicate for each sample. The data are reported as the mean ± SD. * indicates p < 0.05 compared to the Con group. Abbreviations: Con, Control group; *Lep* KO, *Leptin* knockout mice; HFD, High fat diet; RT-qPCR, Quantitative real time-PCR; MUC2, Mucin 2; AQP, Aquaporin.

The changes in the levels of mucin secretion and water channel expressions were measured in the mid colon of the *Lep* KO and HFD-treated mice to determine if the decreases in mucin secretion ability and water channel expression in the mid colon were similar in both groups. The intensity of the goblet cells stained dark blue was significantly lower in the crypts of Lieberkuhn of the *Lep* KO and HFD-treated mice than in the Con mice. There was no difference between the *Lep* KO and HFD-treated mice (Fig 5A). In addition, mucin staining analysis completely reflected the level of MUC2 mRNA expression in the mid colon. A decrease in the MUC2 mRNA level was detected in the mid colon of *Lep* KO and HFD-treated mice compared to the Con mice, but the decrease rate was slightly higher in the *Lep* KO mice than in the HFD-treated mice (Fig 5A and 5B). Furthermore, the levels of the AQP3 and AQP8 transcripts were 38% and 45% lower in the mid colon of *Lep* KO mice than in the Con mice. A similar pattern was observed in the HFD-treated mice, but the decrease rate was slightly greater than in the *Lep* KO mice (Fig 5B). These results show that the decrease in mucin secretion ability and water channel expression was similar in the mid colon of *Lep* KO and HFD-treated mice, but there was a slight difference.

### Differences in the levels of ICC, nitrergic enteric, and myenteric neurons between the *Lep* KO and HFD-treated mice

**Fig 6:**
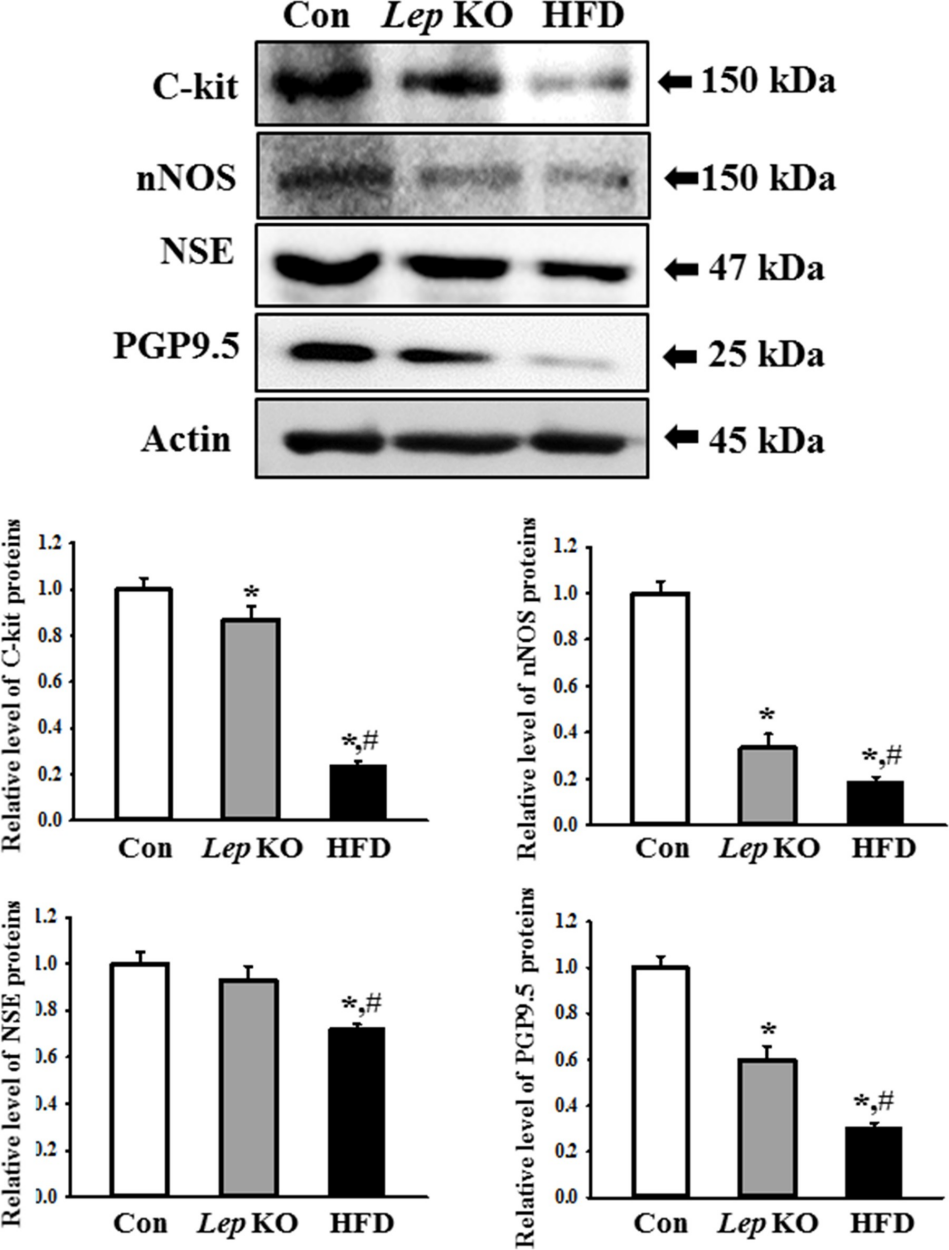
Expression of c-kit, nNOS, NSE, and PGP9.5. The expression levels of four proteins were measured by Western blot analysis using the specific primary antibodies and HRP-labeled anti-rabbit IgG antibody. Three to five mices per group were used to prepare the total tissue homogenate, and Western blot analyses were assayed in duplicate in each sample. The data are reported as the mean ± SD. * indicates p < 0.05 compared to the Con group. Abbreviations: Con, Control group; *Lep* KO, *Leptin* knockout mice; HFD, High fat diet; c-kit, Receptor tyrosine kinase; nNOS, Neuronal nitric oxide synthase; NSE, Neuron-specific enolase; PGP9.5, Protein gene product 9.5.

The expression levels of the markers, including c-kit, nNOS, NSE, and PGP9.5 proteins, were measured in the mid colon of the *Lep* KO and HFD-treated mice to determine if the loss of ICC, nitrergic enteric, and myenteric neurons were similar in both models. The expression levels of the four proteins were significantly lower in the mid colon of the *Lep* KO and HFD-treated mice than in the Con mice, but the decrease rate was different in the two groups. These levels were lower in the HFD-treated mice than in the *Lep* KO mice. In particular, the level of c-kit expression for ICC was 15% and 76% lower in the *Lep* KO and HFD-treated mice, respectively, than in the Con mice (Fig 6). In addition, the level of nNOS expression for the nitrergic enteric neurons was 67% and 82% lower in the *Lep* KO and HFD-treated mice, respectively, than in the Con mice (Fig 6). Furthermore, a similar decrease in NSE and PGP9.5 expression was detected. These levels were significantly lower in the *Lep* KO and HFD-treated mice than in the Con mice, but the decrease was greater in the HFD-treated mice (Fig 6). Hence, the losses of ICC, nitrergic enteric, and myenteric neurons in the mid colon were greater in the HFD-treated mice than in the *Lep* KO mice.

### Differences in the expression levels of the mAChRs and serotonin receptors between the *Lep* KO and HFD-treated mice

**Fig 7:**
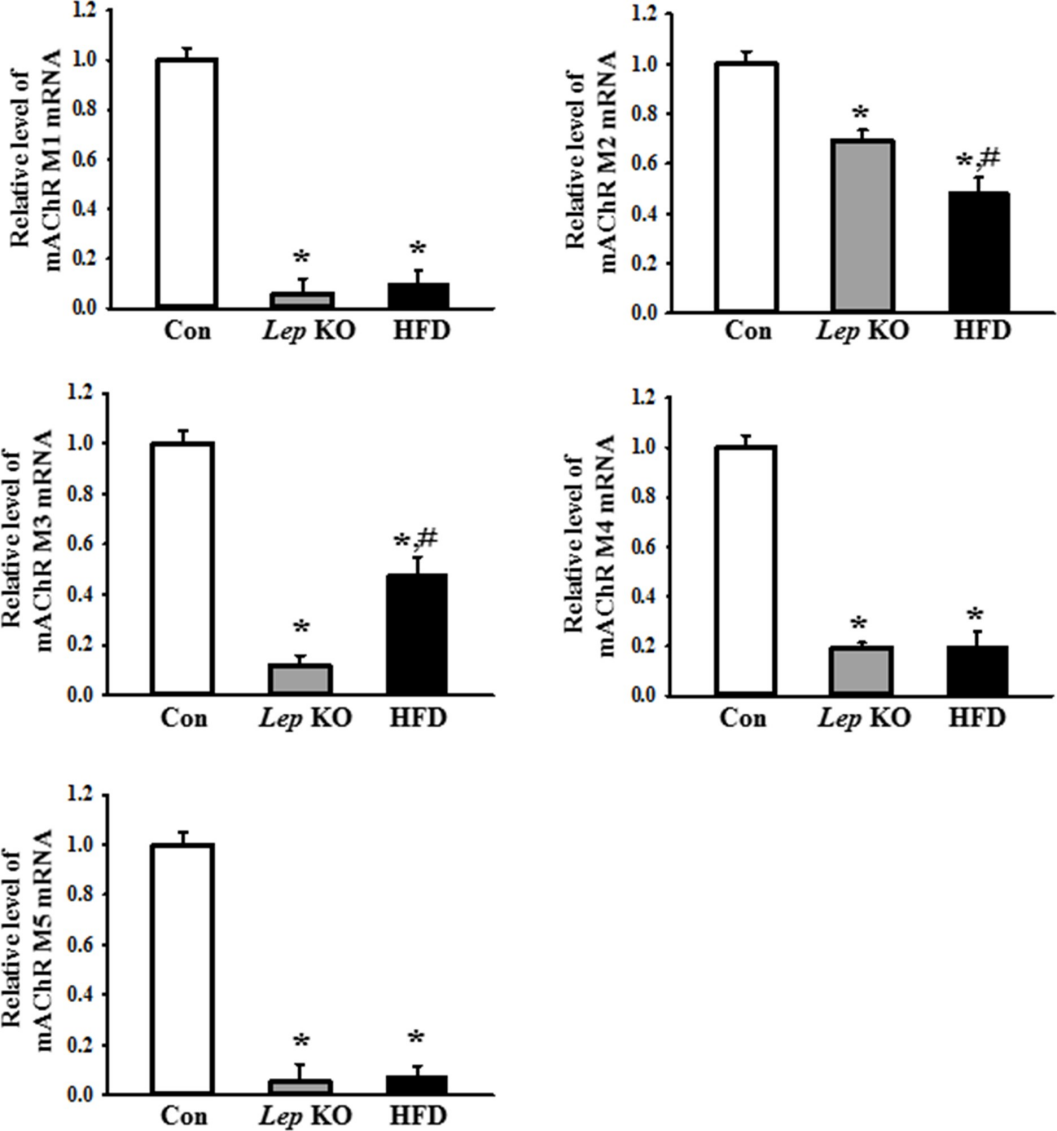
Expression of mAChR M1, M2, M3, M4, and M5 mRNA in the mid colon. The levels of the five mAChRs mRNA in the total mRNA of the mid colon were measured by RT-qPCR using the specific primers. The mRNA levels of these genes were calculated based on the intensity of β-actin as an endogenous control. Three to five mices per group were used to prepare the total RNA. The RT-qPCR analyses were assayed in duplicate for each sample. The data are reported as the mean ± SD. * indicates p < 0.05 compared to the Con group. Abbreviations: Con, Control group; *Lep* KO, *Leptin* knockout mice; HFD, High fat diet; mAChR, muscarinic acetylcholine receptors.

**Fig 8:**
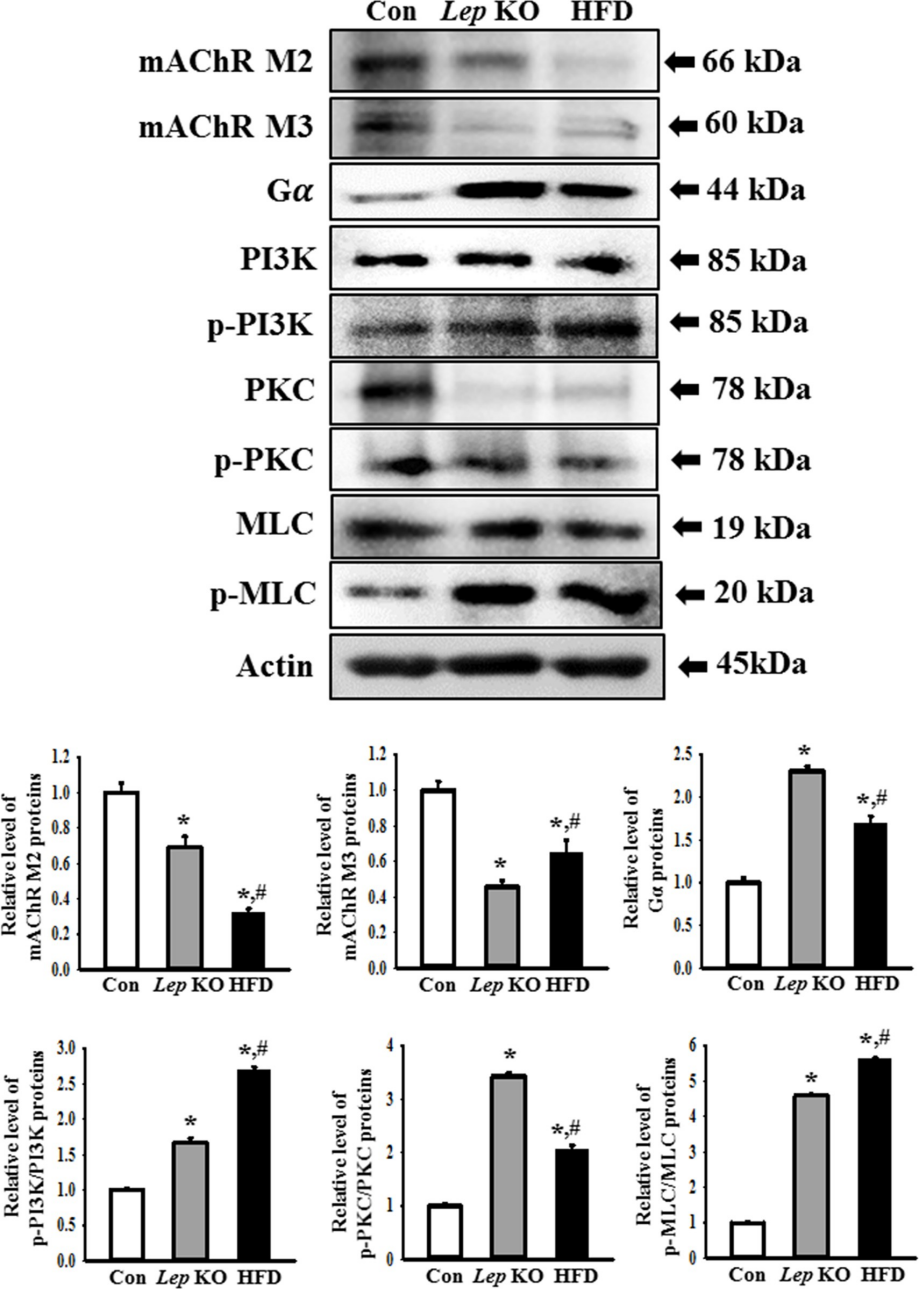
Expression of mAChRs proteins and the key mediators within their downstream signaling pathway. Expression levels of mAChR M2/M3 and the key mediators, including Gα, PKC, p-PKC, PI3K, p-PI3K, MLC, and p-MLC were measured by Western blot analysis using the specific primary antibodies and HRP-labeled anti-rabbit IgG antibody. The intensity of each lane for a specific protein was calculated based on the intensity of β-actin. Three to five mices per group were used to prepare the total tissue homogenate, and Western blot analyses were assayed in duplicate in each sample. The data are reported as the mean ± SD. * indicates p < 0.05 compared to the Con group. Abbreviations: Con, Control group; *Lep* KO, *Leptin* knockout mice; HFD, High fat diet; mAChR, muscarinic acetylcholine receptors; PKC, Protein kinase C; PI3K, Phosphoinositide 3-kinases; MLC, Myosin light chain.

**Fig 9:**
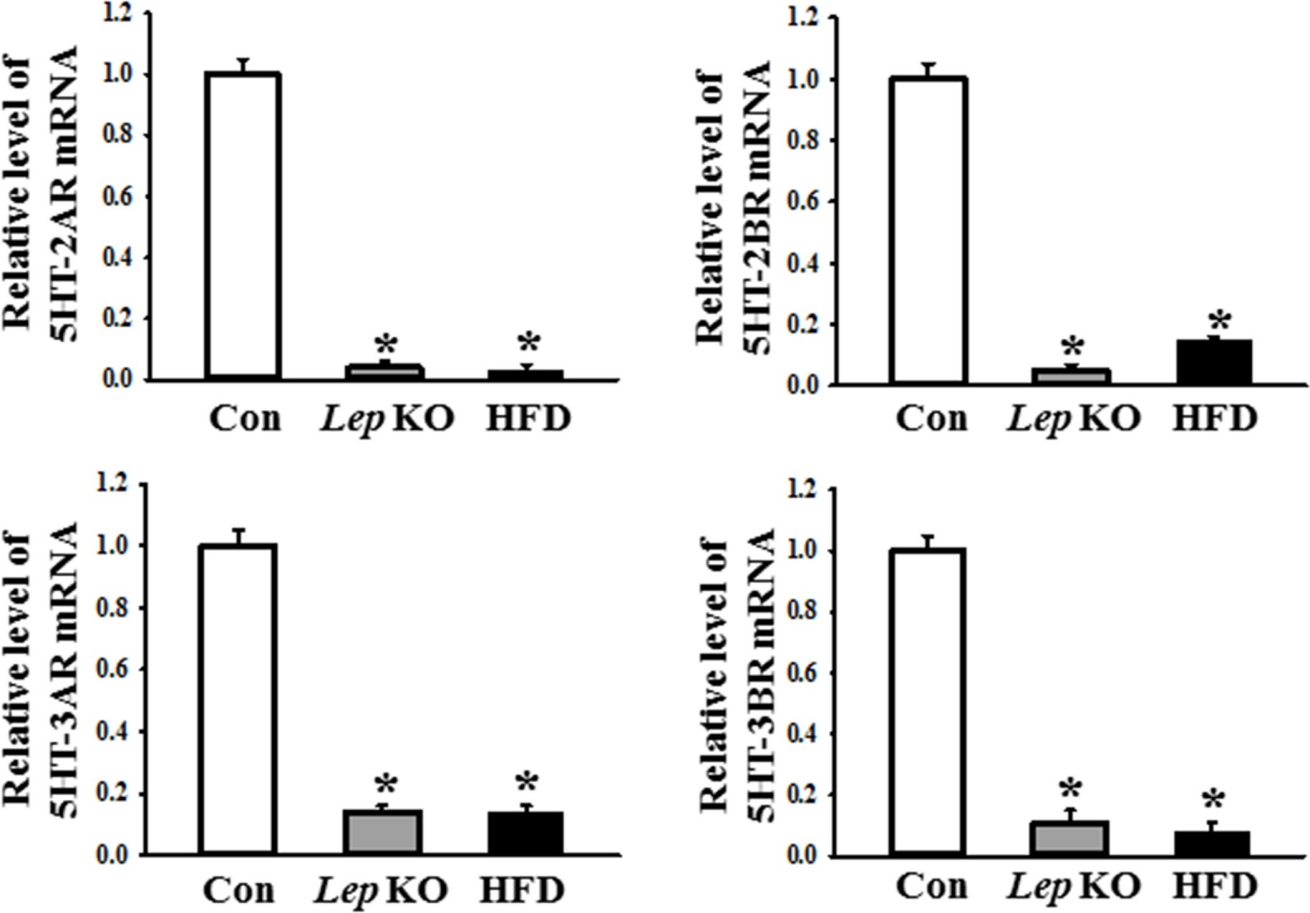
Expression of 5HT-2AR, 2BR, 3AR, and 3BR mRNA in the mid colon. The levels of four serotonin receptors mRNA in the total mRNA of the mid colon were measured by RT-qPCR using the specific primers. The mRNA levels of three genes were calculated based on the intensity of β-actin as an endogenous control. Three to five mices per group were used to prepare the total RNA. The RT-qPCR analyses were assayed in duplicate for each sample. The data are reported as the mean ± SD. * indicates p < 0.05 compared to the Con group. Abbreviations: Con, Control group; *Lep* KO, *Leptin* knockout mice; HFD, High fat diet.

Finally, this study examined whether the changes in the expression levels of mAChRs and the serotonin receptors in the *Lep* KO mice were detected similarly in the HFD-treated mice. The expression levels of mAChR M1, 2, 3, 4, and 5 mRNA, as well as 5HT-2AR, 2BR, 3AR, and 3BR mRNA, were measured in the mid colons of a subset of the groups. The levels of five mAChRs were lower in the mid colon of *Lep* KO and HFD-treated mice than in the Con mice. Among these, mAChR M1, M4, and M5 showed an extremely low expression level, while mAChR M2 and M3 exhibited an intermediate level in the same group (Fig 7). Based on these changes, the changes in the downstream signaling pathway of mAChRs were examined further to confirm whether changes in receptor expression are reflected in their signal pathway. Several significant changes in mAChR M2, mAChR M3, Gα, PKC, p-PKC, PI3K, and p-PI3K expression were observed. The expression levels of the mAChR M2 and M3 proteins were significantly lower in the mid colon of the *Lep* KO and HFD-treated mice than in the Con mice. On the other hand, the level of mAChR M2 expression was lower in the HFD-treated mice, whereas the level of mAChR M3 expression was lower in the *Lep* KO mice (Fig 8). An opposite pattern was observed in the expression and phosphorylation levels of the key members in the downstream signaling pathway of mAChRs. The increases in Gα expression and PKC phosphorylation were greater in the *Lep* KO mice than in the HFD-treated mice, while the increases in PI3K and MLC phosphorylation were greater in the HFD-treated mice than in the *Lep* KO mice (Fig 8). The expression of serotonin receptors showed a similar pattern of change to the expression of mAChRs. The expression levels of 5HT-2AR, 2BR, 3AR, and 3BR were significantly lower in the mid colon of the *Lep* KO and HFD-treated mice than in the Con mice (Fig 9). These results show a decrease in mAChRs and serotonin receptors expression and upregulation in the downstream signal pathway of mAChRs in the mid colon of two animals, but their rate of change is different.

## Discussion

Constipation is induced by various primary causes, including colorectal dysfunction, slow transit, dyssynergic defecation, and irritable bowel syndrome (IBS), as well as secondary causes, such as endocrine disorders, neurogenic disorders, medications, and multiple environments [21]. The similarities and differences in phenotypes and action mechanisms based on the cause of constipation are important for identifying treatments and targets for this disease [22]. As an ongoing part of these studies, this study compared the phenotypes for constipation in the gene defect-induced obesity model and diet-induced obesity model to examine the similarities and differences between the two models. The results provide the first scientific evidence that the two obesity models have overall similar constipation phenotypes, such as excretion parameters, GI transit, histological structure of colon, and mucin secretion ability, but a significant difference in only the ENS regulatory factors, including the ICC number, neuron number, mAChRs expression, and their downstream signaling pathway, and serotonin receptors expression. Hence, the model animal should be considered carefully when studying the regulation of the ENS and mAChR signaling pathways for constipation.

Many rodent and nonrodent models have been used in mechanism studies and evaluations of therapeutic drugs for obesity because they exhibit human-like phenotypes [23]. Among these, the gene-defect and diet-induced models for mice have been applied widely as important animal models for obesity. Ob/ob mice with a *Lep* gene mutation were discovered as obese mice at the Jackson Laboratory in 1949 [24]. They showed unmanageable food intake, obesity, diabetes, and insulin resistance. In addition, the body weight tripled compared to the control group [25]. The db/db mice with a mutation in the *Lep* receptor gene exhibit a defect of the *Lep* receptor downstream signaling pathway. This defect leads to increased fat deposition, hyperglycemia, hyperphagia, insulin resistance, and diabetes [26, 27]. On the other hand, a HFD-induced obesity model in the C57BL/6 strain is a well-known non-*Lep*-deficient model. These mice exhibit similar significant phenotypes to obesity in humans [28, 29]. Furthermore, the blood glucose and serum insulin levels were determined in both animal models. The *Lep* KO mice produced by the CRISPR-Cas9 system exhibited high blood glucose (130–150 mg/dL) and insulin (8–20 ng/mL) levels at 14-week-old after 16 h fasting [30]. At 20 weeks of age, these levels in the *Lep* KO mice changed remarkably to 252–275 mg/dL and 6–7.5 ng/mL, respectively, even though there were some differences according to their region [31]. A similar pattern was observed in the HFD-treated mice. After HFD feeding for 12 weeks, the blood glucose and serum insulin concentrations were 130–160 mg/dL and 27–39 μU/mL, respectively [32–34]. In this study, the *Lep* KO and HFD-treated mice were used to compare the phenotypes of constipation. These two models of obesity exhibited similar levels for the basic factors of their phenotypes, including body weight, fat weight, glucose concentration, and histopathological features of fat and liver. On the other hand, they showed significant differences in the expression and phosphorylation of lipogenic proteins, such as ATGL, perilipin, and HSL, as well as LEP expression. The differences between animal models can be attributed to different methods of inducing obesity.

The gender variances in the obesity and constipation phenotypes were different in the *Lep* KO and HFD-treated mice. The *Lep* KO mice did not show significant gender differences in the obesity phenotypes, even though they were not investigated in the constipation phenotypes [30]. The levels of weight gain, hyperglycemia, and hyperinsulinemia were similar in both males and females of *Lep* KO mice, while significant differences are observed in only the metabolism associated with insulin signaling and lipid metabolism [30, 35, 36]. These differences have been investigated better in HFD-treated mice. Male and female mice exhibit significant differences in hyperglycemia, hyperinsulinemia, hyperleptinemia, hypertriglyceridemia, hypercholesterolemia, and insulin resistance [37, 38]. In addition, remarkable gender differences were observed in constipation phenotypes of the HFD-treated mice. GI transit and gastric emptying were increased in male HFD-treated mice, while the number of EC cells, and mucosal 5-HT or 5-HT3 receptor levels were maintained similarly in both sexes of HFD-treated mice [39]. On the other hand, this study has some limitations. Gender differences were not examined because of institutional ethical policies to reduce the unnecessary number of animals.

In many studies on the molecular mechanism and drug development for constipation, several parameters, including the excretion parameters, GI transit, the histopathological structure of the colon, and mucin secretion ability, were considered the key indicators for constipation [40–46]. A study examining the novel causes of constipation reported that the levels of these factors were significantly lower in the *Lep* KO and C3 KO mice with the constipation phenotypes despite the difference in the rate of decrease [17, 47]. A similar decreasing pattern in constipation was detected in a Tg2576 mice model with the Alzheimer’s disease (AD) phenotypes [45]. In addition, these factors were decreased significantly in the chemically induced constipation models treated with Lop, clonidine, morphine, opioid receptor antagonist, clozapine, and carbon [40, 48–52]. Furthermore, the visceral hypersensitivity was considered as another core pathophysiological mechanism associated with the constipation phenotypes because it may be associated with the pathogenesis of abdominal pain/discomfort and be derived from the sensitization of the nerve afferent pathways originating from the GI tract [53, 54]. In particular, significant changes in the constipation parameters were detected in the HFD-induced obesity model. The fecal weight and calories were lower in the NSY/HOS mice with obesity induced by four weeks of HFD, while the decrease in stool parameters, increase in GI transit time, and decrease in the colonic mucus level were observed in the C57BL/6 mice treated with a HFD for eight weeks [8, 9]. The number of nNOS myenteric neurons and enterochromaffin (EC) cells and the 5-HT concentration were lower in the Western diet (WD)-treated C57BL/6 mice and rats [7, 16]. The present study analyzed the key parameters for constipation in the *Lep* KO and HFD-treated mice compared to the pathological phenotypes between the gene defect model and HFD induced model. Significant changes in most parameters, including stool factors, GI transit, intestine length, histopathological structure, and mucin secretion, were detected similarly in the *Lep* KO and HFD-treated mice. The present results are consistent with previous studies that used *Lep* KO and HFD-treated models despite some differences in the analytical factors.

Complex collaboration and communication of various ENS cells, including enteric neurons, ICC, and smooth muscle, play a crucial role in regulating the gut motility [55]. Therefore, the number of enteric neurons and ICC were changed significantly in the animal models and human patients with constipation. In STC patients, the number of ICC and PGP9.5 reactive cells were remarkably lower in the sigmoid colon or all colonic regions, while a similar decrease in the number of enteric neurons was detected in the submucosal plexus of a patient with intractable constipation [56–59]. In animal models with constipation, the expression levels of the NSE, PGP9.5, and c-kit markers for ENS cells were lower in the mid colon of the *Lep* KO mice and Lop-treated animals than in the control group, but the rate of decrease varied [17, 45, 60]. In the present study, the expression levels of the ENS cell markers in the *Lep* KO and HFD-treated mice were compared to determine if the loss of ICC, nitrergic enteric, and myenteric neurons were similarly detected in obesity-induced constipation models. The expression levels of the four markers were lower in the *Lep* KO and HFD-treated mice than in the Con group, even though they remained higher in the *Lep* KO mice than in the HFD-treated mice. Most of the present results for the loss of enteric neurons and ICC examined in the *Lep* KO and HFD-treated mice concurred with previous studies that reported a decrease in the enteric neurons and ICC in the patient and animal models showing the constipation phenotypes. On the other hand, the decreasing pattern in these markers did not coincide with the decreasing pattern in the GI motility. This difference is believed to be important in the loss of different types of cells in each model animal. The loss of nitrergic enteric neurons may affect the suppression of GI motility in *Lep* KO mice, but the loss of ICCs and myenteric neurons can significantly affect their decrease in HFD-treated mice because ICCs play a key role during glucose homeostasis [61].

On the other hand, this reports the first study comparing the alterations in the expression level of the markers for ICC, nitrergic enteric, and myenteric neurons in an obesity-induced constipation model. Significant changes in the levels of mAChRs expression and their downstream signaling pathway were detected in *Lep* KO and HFD-treated mice, as shown in Fig 8. On the other hand, their alteration rates were different in the *Lep* KO and HFD-treated mice. These changes indicate that obesity-induced constipation may be tightly linked to regulating the ACh neurotransmitter. Similar results on the mAChR signaling pathway have been detected in several animal models with constipation phenotypes. The decrease in the mAChR M2 and M3 expression level and activation of their downstream signaling pathway was observed in a Lop-induced constipation rat and Tg2576 mice [42, 44, 45]. Furthermore, a microplastics (MP)-induced constipation model showed a significant change in mAChRs expression and their downstream signaling pathway, despite the difference in the level of change [19]. Furthermore, a decrease in AChE activity was detected in the colon tissue of Lop-induced constipation rats and serum of activated carbon-induced constipation mice [34, 62]. Nevertheless, the difference in the alteration rate of mAChR expression and their downstream signaling pathway between each constipation model is unknown because there has been no direct comparison until now.

## Conclusions

This study compared the phenotypes for constipation of *Lep* KO mice with those of HFD-treated mice to determine if the phenotypes for constipation in the *Lep* KO mice were detected similarly in HFD-treated mice. Most general markers for constipation were similar in the *Lep* KO and HFD-treated mice, but several markers for the regulatory function of ENS showed a different response (Table 1). Furthermore, these findings provide scientific evidence that the regulatory mechanism of ENS during chronic constipation should be considered an important factor in selecting an obesity-induced constipation model. However, this study had some limitations in that it did not comprehensively investigate any of the pathways potentially regulated by obesity, only the expression level of several members of each pathway, and only at a single timepoint because obesity-related constipation pathways are much more complex. In addition, the lack of comparison in relation to other obesity animal models with different induction mechanisms should be considered a limitation of the present study.

**Table 1:** Similarities and differences in obesity and constipation phenotypes between the *Lep* KO and HFD-treated mice. The minus sign indicates a decrease compared to the Con mice, and the plus sign indicates an increase in the same condition.

Categories			Relative level of change compared to Con group	
			*Lep* KO mice (%)	HFD-treated mice (%)
Obesity phenotype	Bodyweight		77.9±3.5	63.7±2.8
	Epididymal Fat weight		353.1±23.4	291.6±16.7
	Retroperitoneal fat weight		528.5±11.1	443.4±17.3
	Lep expression		-30.1±2.3	130.3±16.7
	Glucose concentrations		262.3±15.1	145.3±16.1
	Histological structure	Adipocyte size in fat	410.9±21.9	370.6±18.5
		Adipocyte number in liver	448.0±26.2	169.0±13.6
	Lipogenic proteins	ATGL	-33.6±5.2	13.3±3.3
		Perilipin	1358±55.8	34.62±36.2
		HSL	308.0±33.0	-52.6±4.7
Constipation phenotype	Excretion parameters	Stool number	-78.6±9.2	-76.4±7.2
		Stool weight	-81.7±5.2	-80.7±6.8
		Stool water content	-42.6±6.5	-53.3±7.6
		Urine volume	34.5±4.3	35.7±2.9
		Food intake	34.4±4.8	24.1±6.3
		Water consumption	-6.8±1.3	-2.2±1.3
	GI transit	Transit ratio	-28.8±9.8	-22.5±1.3
		Intestinal length	-9.9±2.2	-12.6±2.0
	Histological structure	Mucosa layer thickness	-41.8±1.6	-38.9±5.8
		Muscle thickness	-47.4±7.3	-31.1±5.1
	Mucin secretion	MUC2 mRNA level	-32.4±4.3	-25.5±3.3
		AQP3 mRNA level	-38.2±2.5	-52.4±6.8
		AQP8 mRNA level	-45.3±5.5	-57.4±7.2
	ENS composition	c-kit level	-13.1±0.9	-76.1±2.6
		nNOS level	-66.7±3.3	-81.1±8.1
		NSE level	-7.1±0.9	-27.7±2.1
		PGP9.5 level	-40.1±5.9	-69.3±6.1
	ENS regulation	mAChR M1 mRNA	-94.0±4.3	-89.6±7.1
		mAChR M2 mRNA	-30.5±3.2	-51.4±3.1
		mAChR M3 mRNA	-88.2±8.4	-52.2±4.1
		mAChR M4 mRNA	-80.5±4.8	-80.0±6.8
		mAChR M5 mRNA	-94.6±8.4	-92.4±6.4
		mAChR M2 proteins	-30.8±1.8	-67.6±3.6
		mAChR M3 proteins	-54.5±4.2	-35.2±7.2
		Gα level	131.1±11.3	69.9±9.8
		PI3K phosphorylation level	67.59±5.6	170.0±27.4
		PKC phosphorylation level	242.8±28.6	105.3±12.6
		MLC phosphorylation level	358.3±25.3	463.2±32.2
		5HT-2AR mRNA	-96.2±6.4	-97.1±6.7
		5HT-2BR mRNA	-95.1±4.8	-85.8±6.1
		5HT-3AR mRNA	-86.4±3.8	-86.1±4.1
		5HT-3BR mRNA	-89.3±4.8	-92.6±3.4

## Supporting information

S1 Fig Spearman correlation between body weight and stool parameters in *Lep* KO mice. Stools were collected from *Lep* KO mice bred in a metabolic cage. The statistical analysis for the correlation between the body weight and the stool number/water contents was performed by the Spearman’s rank correlation method.
(TIF)

S1 Raw images (PDF)
