# Supplementary figures and images for: Similarities and differences in constipation phenotypes between Lep knockout mice and high fat diet-induced obesity mice
Source: PLoS One. 2022 Dec 22;17(12):e0276445. doi: 10.1371/journal.pone.0276445 (PMC9778951; doi:10.1371/journal.pone.0276445)

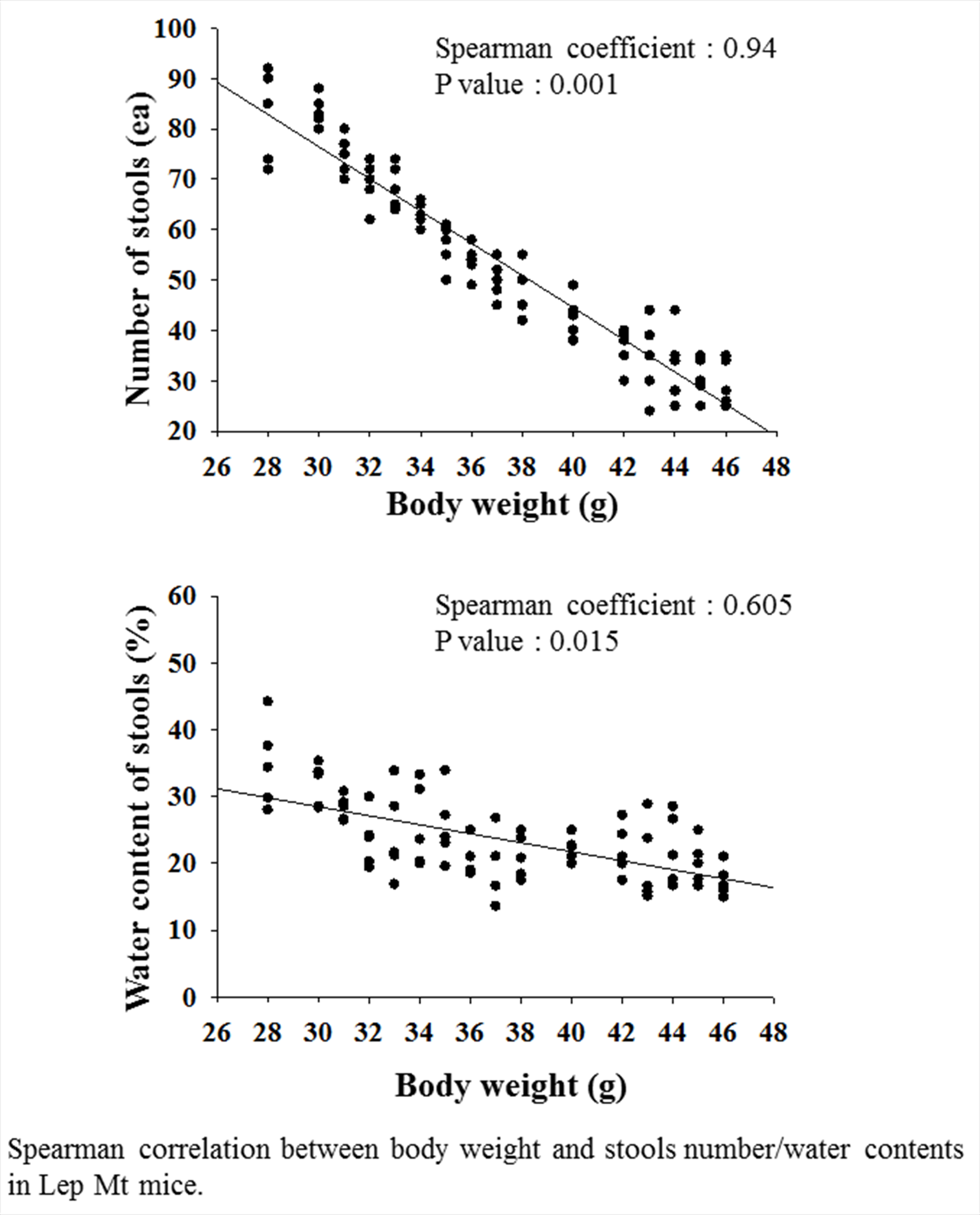

Supplement: S1 Fig — Stools were collected from Lep KO mice bred in a metabolic cage. The statistical analysis for the correlation between the body weight and the stool number/water contents was performed by the Spearman’s rank correlation method. (TIF) [file pone.0276445.s001.tif]
