# Supplementary material for: Similarities and differences in constipation phenotypes between Lep knockout mice and high fat diet-induced obesity mice
Source: PLoS One. 2022 Dec 22;17(12):e0276445. doi: 10.1371/journal.pone.0276445 (PMC9778951; doi:10.1371/journal.pone.0276445)
Supplement: S1 Raw images — (PDF) [file pone.0276445.s002.pdf]

Supporting Materials 1

Original Western blot image for ATGL proteins in Fig. 1

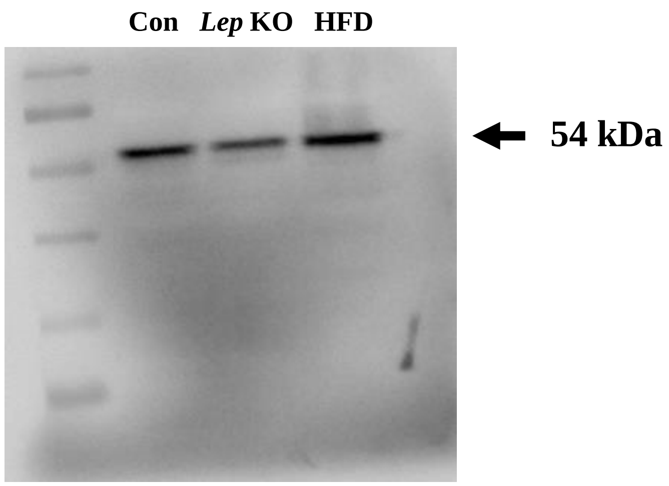

Expression of ATGL in the liver tissue of mice. Briefly, total lysates of liver were prepared using homogenizer and separated in SDS-PAGE gel. The expression level of actin protein with colon homogenate transferred on the membrane was determined by HRP-conjugated anti-rabbit IgG antibody during Western blot analysis.

Supporting Materials 2

Original Western blot image for Perilipin proteins in Fig. 1

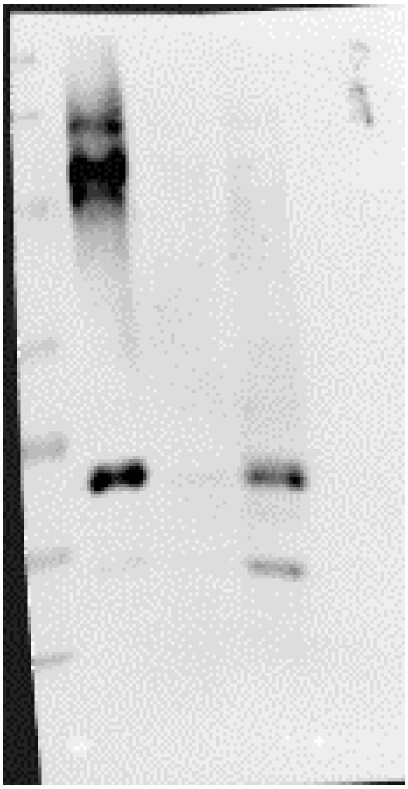

Expression of Perilipin in the liver tissue of mice. Briefly, total lysates of liver were prepared using homogenizer and separated in SDS-PAGE gel. The expression level of actin protein with colon homogenate transferred on the membrane was determined by HRP-conjugated anti-rabbit IgG antibody during Western blot analysis.

Supporting Materials 3

Original Western blot image for p-Perilipin proteins in Fig. 1

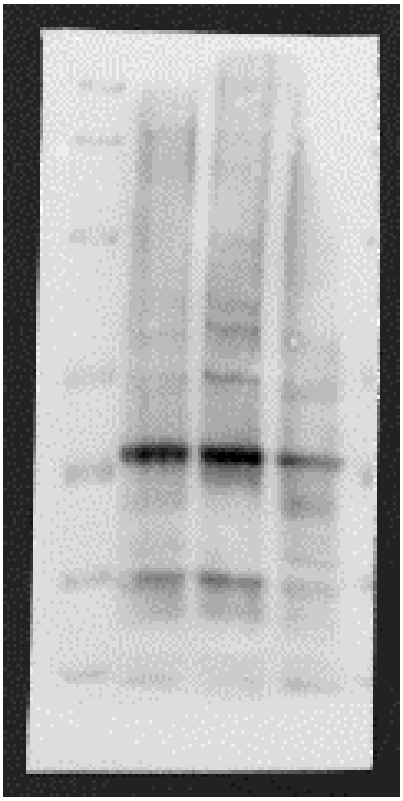

Expression of p-Perilipin in the liver tissue of mice. Briefly, total lysates of liver were prepared using homogenizer and separated in SDS-PAGE gel. The expression level of actin protein with colon homogenate transferred on the membrane was determined by HRP-conjugated anti-rabbit IgG antibody during Western blot analysis.

Supporting Materials 4

Original Western blot image for HSL proteins in Fig. 1

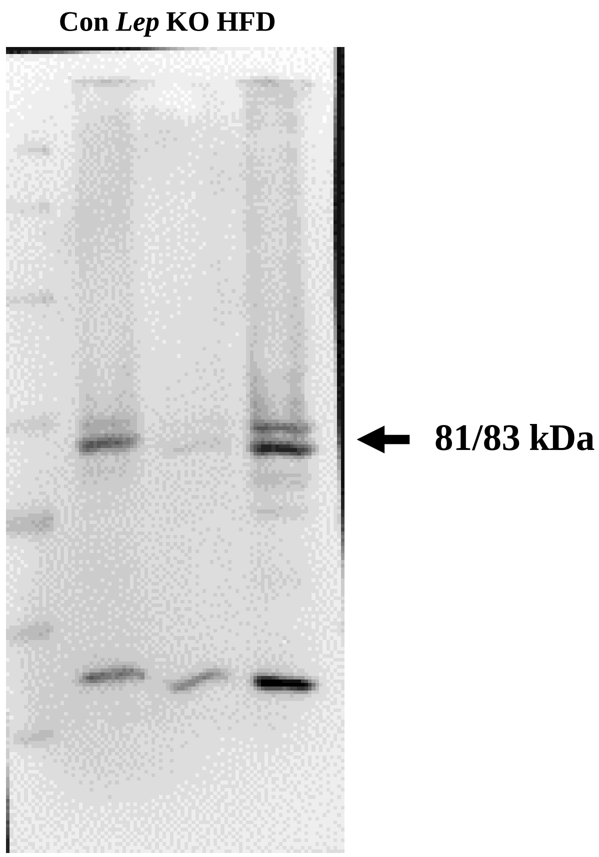

Expression of HSL in the liver tissue of mice. Briefly, total lysates of liver were prepared using homogenizer and separated in SDS-PAGE gel. The expression level of actin protein with colon homogenate transferred on the membrane was determined by HRP-conjugated anti-rabbit IgG antibody during Western blot analysis.

Supporting Materials 5

Original Western blot image for p-HSL proteins in Fig. 1

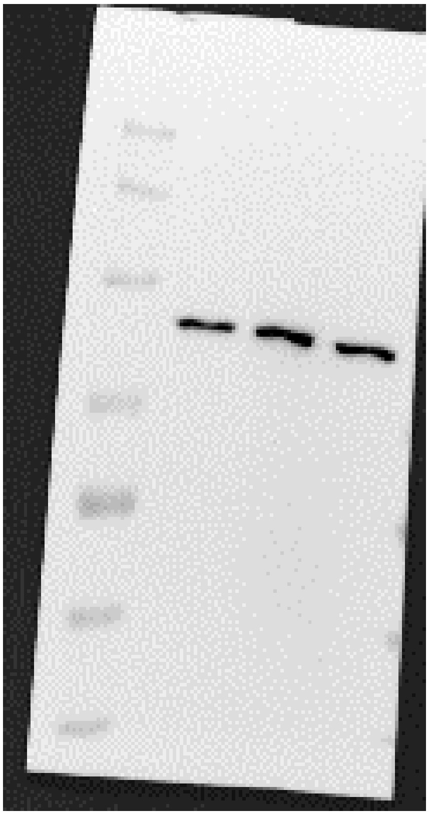

Expression of p-HSL in the liver tissue of mice. Briefly, total lysates of liver were prepared using homogenizer and separated in SDS-PAGE gel. The expression level of actin protein with colon homogenate transferred on the membrane was determined by HRP-conjugated anti-rabbit IgG antibody during Western blot analysis.

Supporting Materials 6

Original Western blot image for Actin proteins in Fig. 1

45 kDa

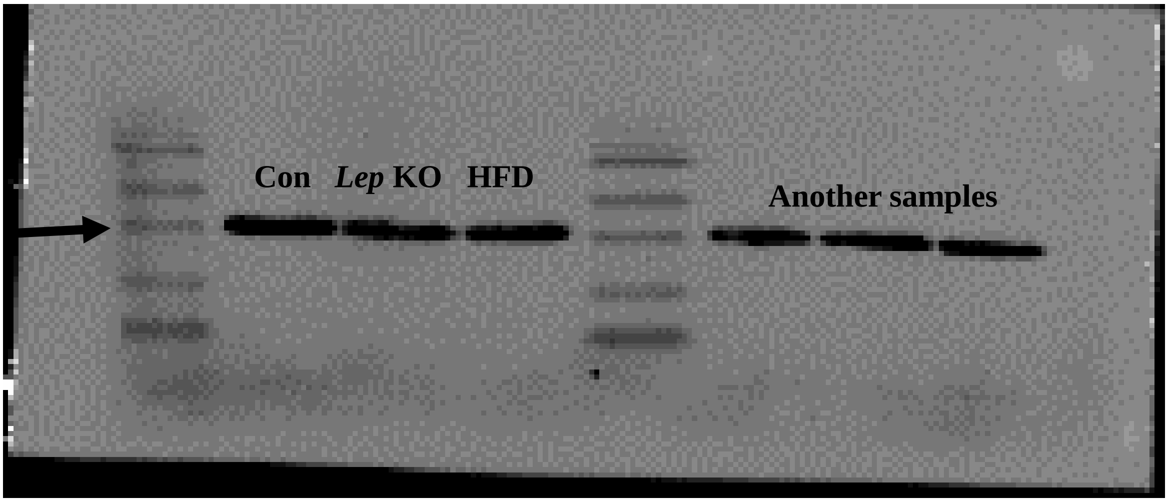

Expression of Actin in the liver tissue of mice. Briefly, total lysates of liver were prepared using homogenizer and separated in SDS-PAGE gel. The expression level of actin protein with colon homogenate transferred on the membrane was determined by HRP-conjugated anti-rabbit IgG antibody during Western blot analysis.

Supporting Materials 7

Original Western blot image for C-kit proteins in Fig. 6

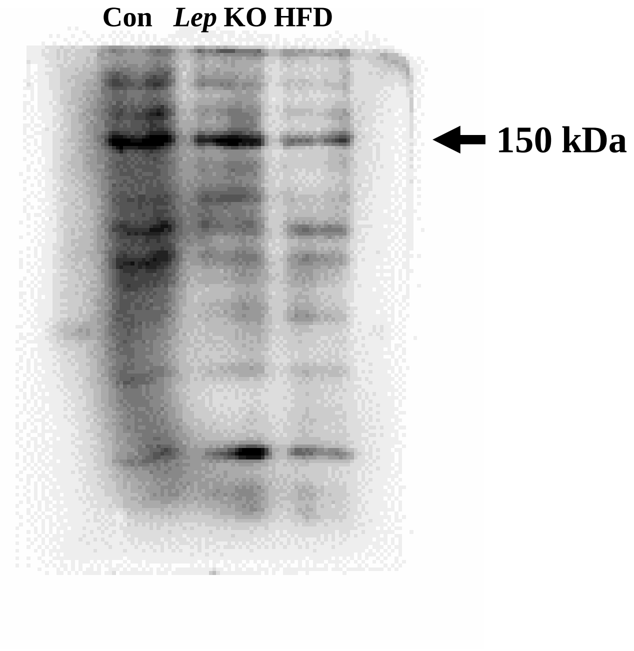

Expression of C-kit in the colon tissue of mice. Briefly, total lysates of colon were prepared using homogenizer and separated in SDS-PAGE gel. The expression level of actin protein with colon homogenate transferred on the membrane was determined by HRP-conjugated anti-rabbit IgG antibody during Western blot analysis.

Supporting Materials 8

Original Western blot image for nNOS proteins in Fig. 6

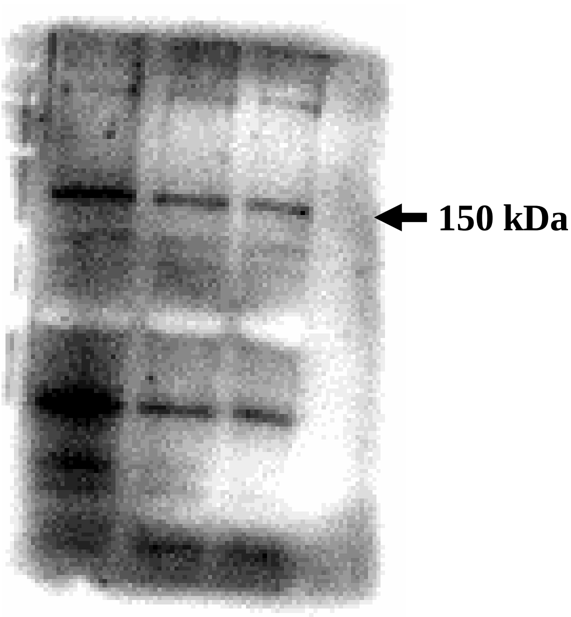

Expression of nNOS in the colon tissue of mice. Briefly, total lysates of colon were prepared using homogenizer and separated in SDS-PAGE gel. The expression level of actin protein with colon homogenate transferred on the membrane was determined by HRP-conjugated anti-rabbit IgG antibody during Western blot analysis.

Supporting Materials 9

Original Western blot image for NSE proteins in Fig. 6

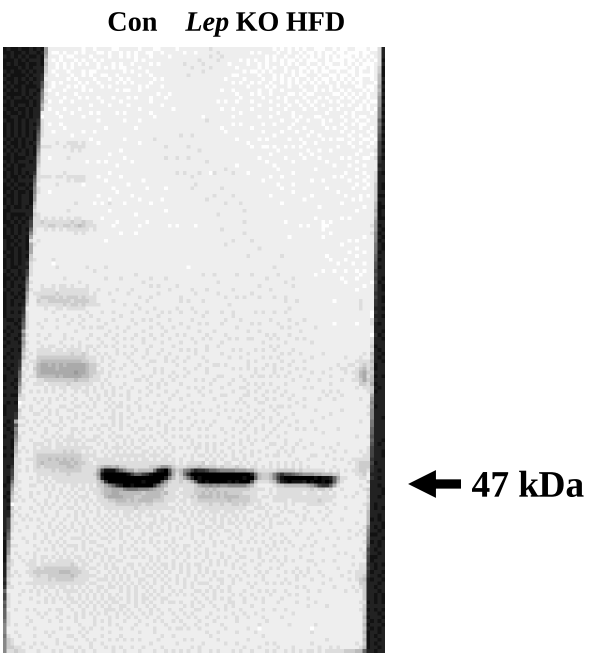

Expression of NSE in the colon tissue of mice. Briefly, total lysates of colon were prepared using homogenizer and separated in SDS-PAGE gel. The expression level of actin protein with colon homogenate transferred on the membrane was determined by HRP-conjugated anti-rabbit IgG antibody during Western blot analysis.

Supporting Materials 10

Original Western blot image for PGP9.5 proteins in Fig. 6

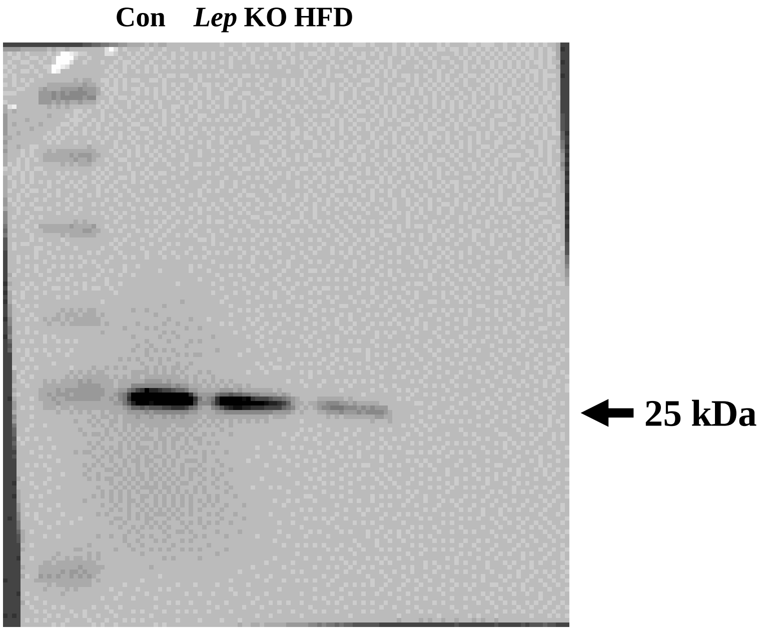

Expression of PGP9.5 in the colon tissue of mice. Briefly, total lysates of colon were prepared using homogenizer and separated in SDS-PAGE gel. The expression level of actin protein with colon homogenate transferred on the membrane was determined by HRP-conjugated anti-rabbit IgG antibody during Western blot analysis.

Supporting Materials 11

Original Western blot image for Actin proteins in Fig. 6

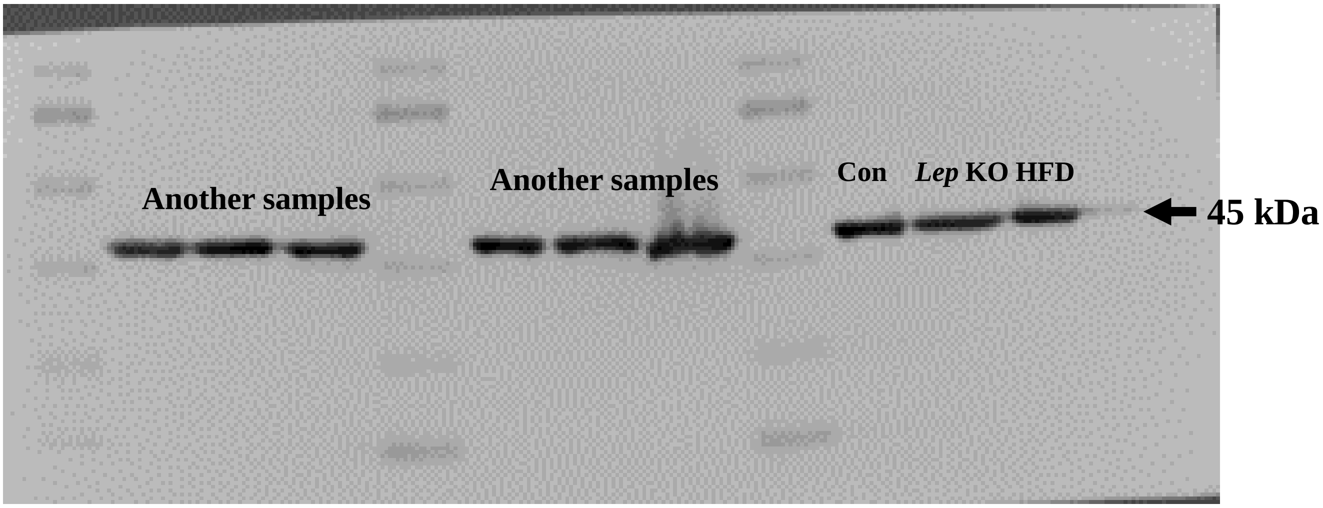

Expression of Actin in the colon tissue of mice. Briefly, total lysates of colon were prepared using homogenizer and separated in SDS-PAGE gel. The expression level of actin protein with colon homogenate transferred on the membrane was determined by HRP-conjugated anti-rabbit IgG antibody during Western blot analysis.

Supporting Materials 12

Original Western blot image for mAChR M2 proteins in Fig. 8

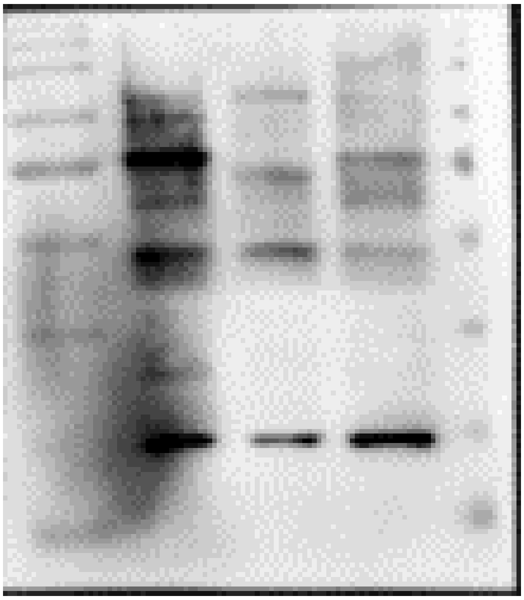

Expression of mAChR M2 in the colon tissue of mice. Briefly, total lysates of colon were prepared using homogenizer and separated in SDS-PAGE gel. The expression level of actin protein with colon homogenate transferred on the membrane was determined by HRP-conjugated anti-rabbit IgG antibody during Western blot analysis.

Supporting Materials 13

Original Western blot image for mAChR M3 proteins in Fig. 8

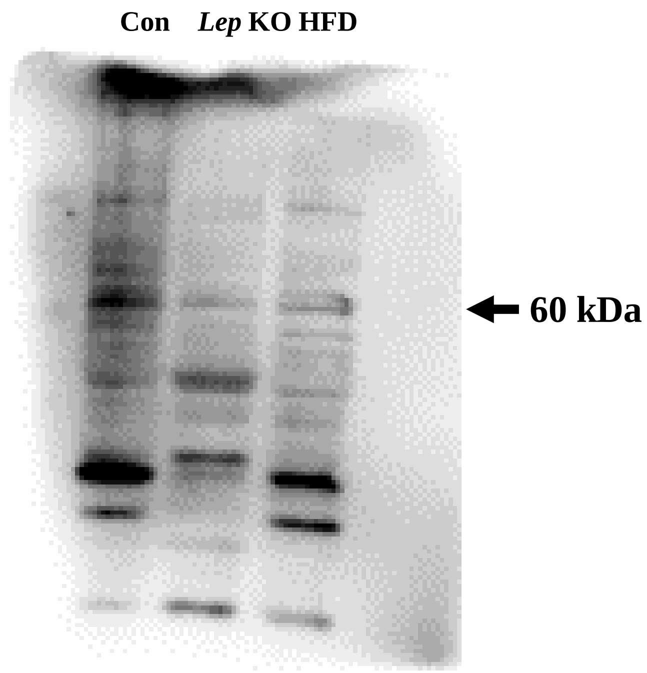

Expression of mAChR M3 in the colon tissue of mice. Briefly, total lysates of colon were prepared using homogenizer and separated in SDS-PAGE gel. The expression level of actin protein with colon homogenate transferred on the membrane was determined by HRP-conjugated anti-rabbit IgG antibody during Western blot analysis.

Supporting Materials 14

Original Western blot image for Gα proteins in Fig. 8

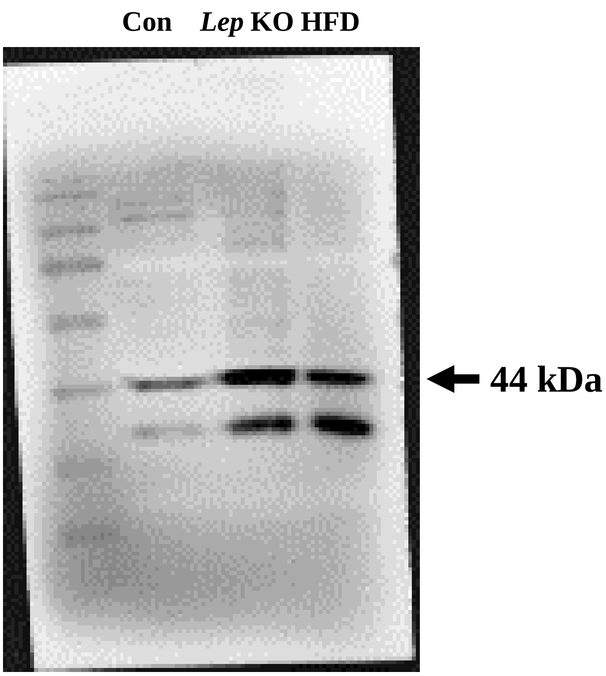

Expression of Gα in the colon tissue of mice. Briefly, total lysates of colon were prepared using homogenizer and separated in SDS-PAGE gel. The expression level of actin protein with colon homogenate transferred on the membrane was determined by HRP-conjugated anti-rabbit IgG antibody during Western blot analysis.

Supporting Materials 15

Original Western blot image for PI3K proteins in Fig. 8

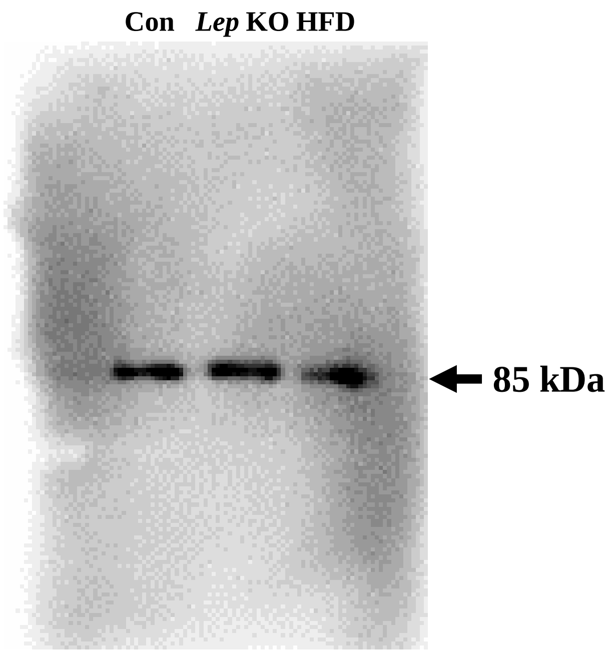

Expression of PI3K in the colon tissue of mice. Briefly, total lysates of colon were prepared using homogenizer and separated in SDS-PAGE gel. The expression level of actin protein with colon homogenate transferred on the membrane was determined by HRP-conjugated anti-rabbit IgG antibody during Western blot analysis.

Supporting Materials 16

Original Western blot image for p-PI3K proteins in Fig. 8

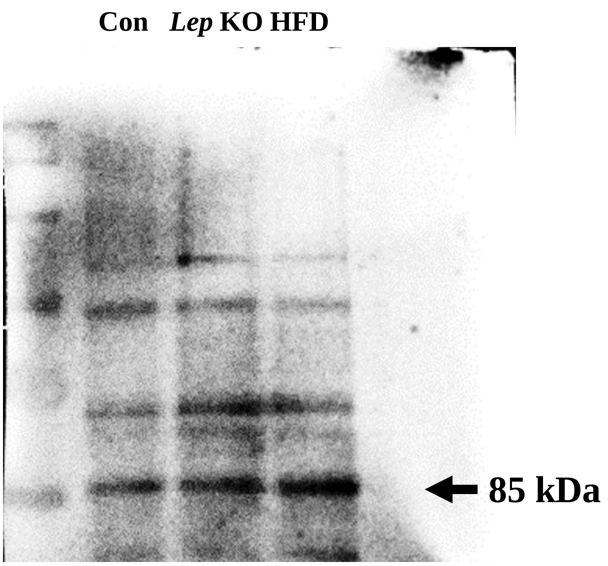

Expression of p-PI3K in the colon tissue of mice. Briefly, total lysates of colon were prepared using homogenizer and separated in SDS-PAGE gel. The expression level of actin protein with colon homogenate transferred on the membrane was determined by HRP-conjugated anti-rabbit IgG antibody during Western blot analysis.

Supporting Materials 17

Original Western blot image for PKC proteins in Fig. 8

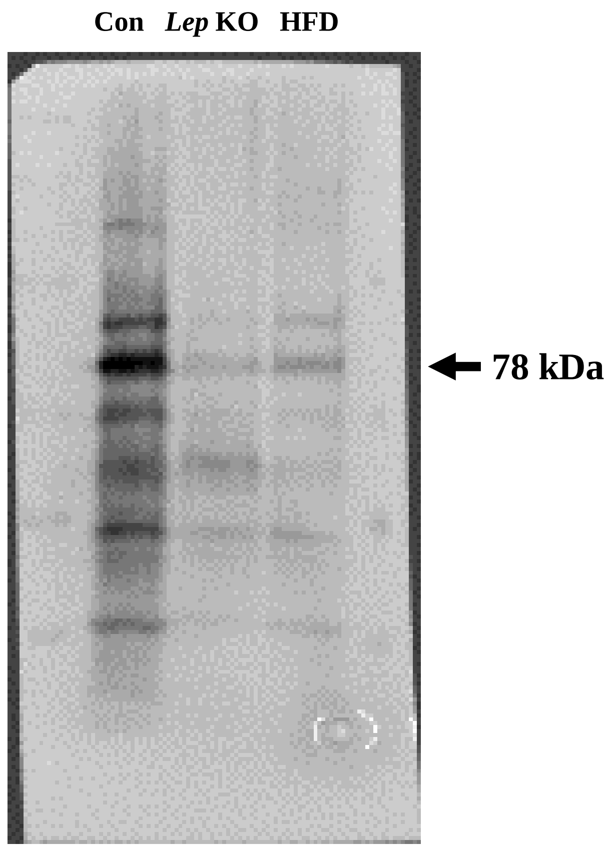

Expression of PKC in the colon tissue of mice. Briefly, total lysates of colon were prepared using homogenizer and separated in SDS-PAGE gel. The expression level of actin protein with colon homogenate transferred on the membrane was determined by HRP-conjugated anti-rabbit IgG antibody during Western blot analysis.

Supporting Materials 18

Original Western blot image for p-PKC proteins in Fig. 8

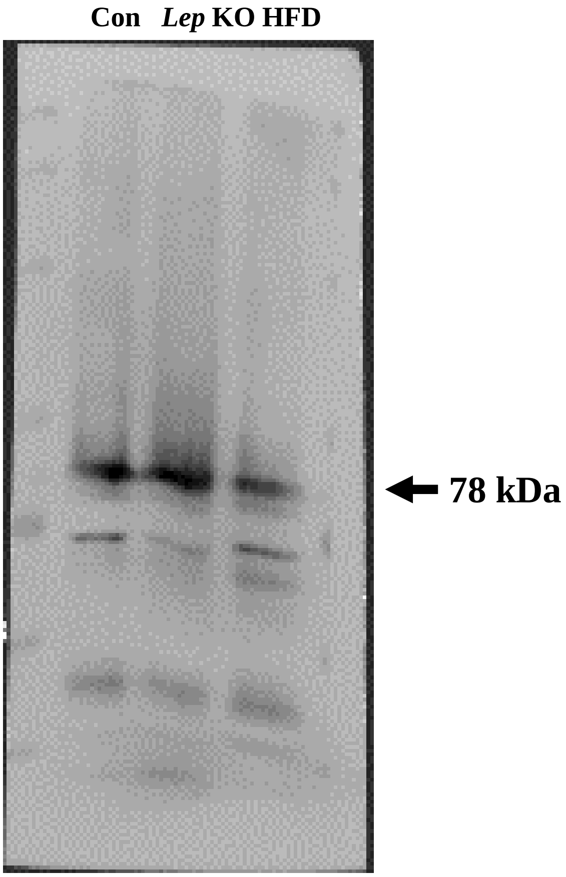

Expression of p-PKC in the colon tissue of mice. Briefly, total lysates of colon were prepared using homogenizer and separated in SDS-PAGE gel. The expression level of actin protein with colon homogenate transferred on the membrane was determined by HRP-conjugated anti-rabbit IgG antibody during Western blot analysis.

Supporting Materials 19

Original Western blot image for MLC proteins in Fig. 8

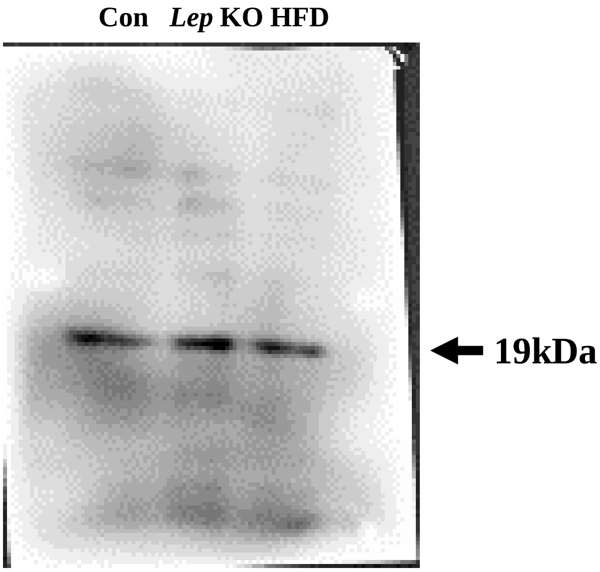

Expression of MLC in the colon tissue of mice. Briefly, total lysates of colon were prepared using homogenizer and separated in SDS-PAGE gel. The expression level of actin protein with colon homogenate transferred on the membrane was determined by HRP-conjugated anti-rabbit IgG antibody during Western blot analysis.

Supporting Materials 20

Original Western blot image for p-MLC proteins in Fig. 8

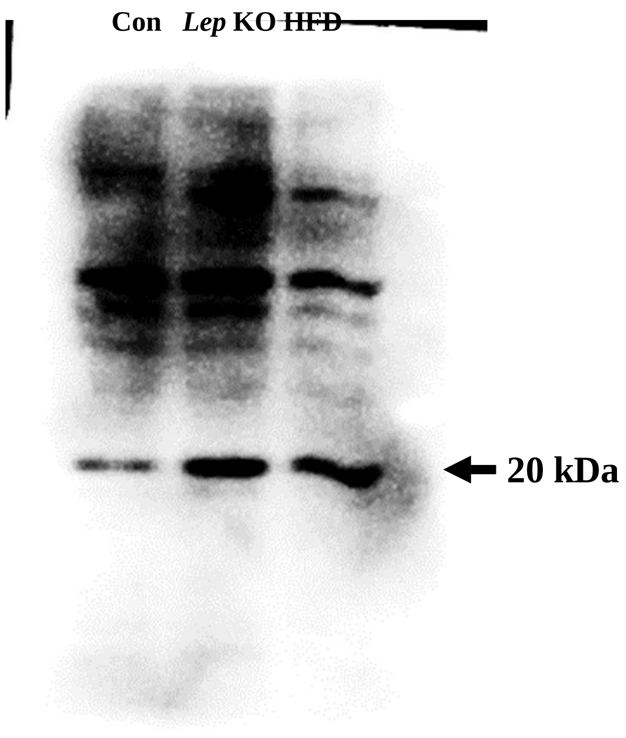

Expression of p-MLC in the colon tissue of mice. Briefly, total lysates of colon were prepared using homogenizer and separated in SDS-PAGE gel. The expression level of actin protein with colon homogenate transferred on the membrane was determined by HRP-conjugated anti-rabbit IgG antibody during Western blot analysis.

Supporting Materials 21

Original Western blot image for Actin proteins in Fig. 8

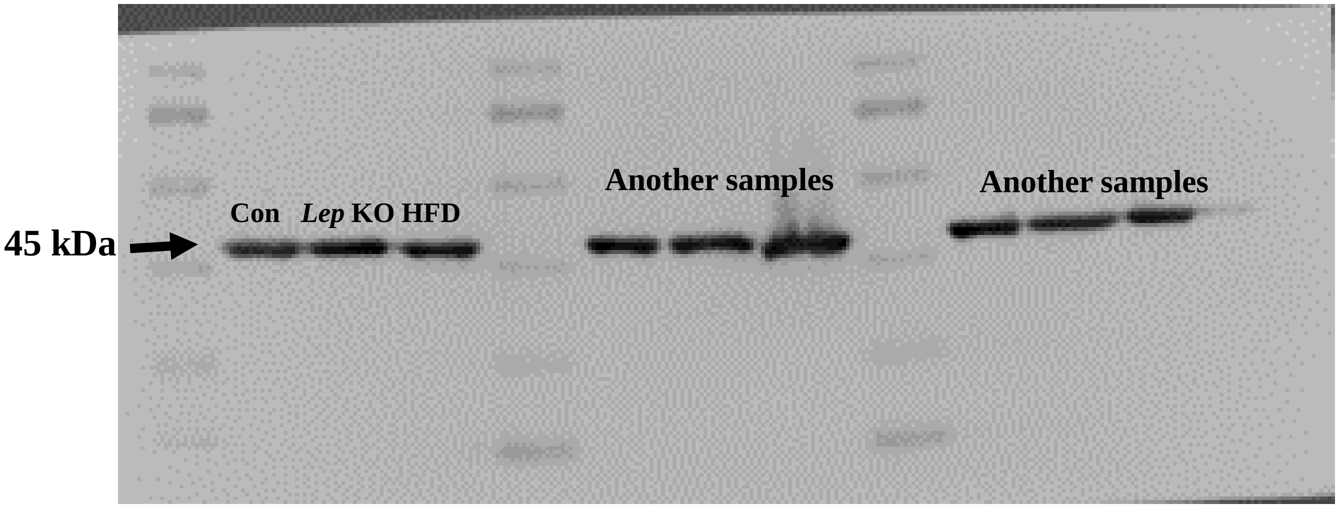

Expression of Actin in the colon tissue of mice. Briefly, total lysates of colon were prepared using homogenizer and separated in SDS-PAGE gel. The expression level of actin protein with colon homogenate transferred on the membrane was determined by HRP-conjugated anti-rabbit IgG antibody during Western blot analysis.
